# Temporal and Spatial Changes in Phyllosphere Microbiome of Acacia Trees Growing in Arid Environments

**DOI:** 10.3389/fmicb.2021.656269

**Published:** 2021-07-12

**Authors:** Ashraf Al Ashhab, Shiri Meshner, Rivka Alexander-Shani, Hana Dimerets, Michael Brandwein, Yael Bar-Lavan, Gidon Winters

**Affiliations:** ^1^Dead Sea and Arava Science Center, Masada, Israel; ^2^Ben-Gurion University of the Negev, Eilat Campus, Be'er Sheva, Israel; ^3^Biofilm Research Laboratory, Faculty of Dental Medicine, Hebrew University of Jerusalem, Jerusalem, Israel

**Keywords:** *Acacia raddiana*, *Acacia tortilis*, phyllosphere, desert plants, microbiome, endophytes, epiphytes

## Abstract

**Background:** The evolutionary relationships between plants and their microbiomes are of high importance to the survival of plants in general and even more in extreme conditions. Changes in the plant's microbiome can affect plant development, growth, fitness, and health. Along the arid Arava, southern Israel, acacia trees (*Acacia raddiana* and *Acacia tortilis*) are considered keystone species. In this study, we investigated the ecological effects of plant species, microclimate, phenology, and seasonality on the epiphytic and endophytic microbiome of acacia trees. One hundred thirty-nine leaf samples were collected throughout the sampling year and were assessed using 16S rDNA gene amplified with five different primers (targeting different gene regions) and sequenced (150 bp paired-end) on an Illumina MiSeq sequencing platform.

**Results:** Epiphytic bacterial diversity indices (Shannon–Wiener, Chao1, Simpson, and observed number of operational taxonomic units) were found to be nearly double compared to endophyte counterparts. Epiphyte and endophyte communities were significantly different from each other in terms of the composition of the microbial associations. Interestingly, the epiphytic bacterial diversity was similar in the two acacia species, but the canopy sides and sample months exhibited different diversity, whereas the endophytic bacterial communities were different in the two acacia species but similar throughout the year. Abiotic factors, such as air temperature and precipitation, were shown to significantly affect both epiphyte and endophytes communities. Bacterial community compositions showed that Firmicutes dominate *A. raddiana*, and Proteobacteria dominate *A. tortilis*; these bacterial communities consisted of only a small number of bacterial families, mainly *Bacillaceae* and *Comamonadaceae* in the endophyte for *A. raddiana* and *A. tortilis*, respectively, and *Geodematophilaceae* and *Micrococcaceae* for epiphyte bacterial communities, respectively. Interestingly, ~60% of the obtained bacterial classifications were unclassified below family level (i.e., “new”).

**Conclusions:** These results shed light on the unique desert phyllosphere microbiome highlighting the importance of multiple genotypic and abiotic factors in shaping the epiphytic and endophytic microbial communities. This study also shows that only a few bacterial families dominate both epiphyte and endophyte communities, highlighting the importance of climate change (precipitation, air temperature, and humidity) in affecting arid land ecosystems where acacia trees are considered keystone species.

## Introduction

The aboveground surfaces of plants (the phyllosphere) harbor a diverse variety of microorganisms, including bacteria (Stone et al., 2018). The microbiome of the plant phyllosphere has been shown to play an important role in the adaptation of the plant host to different environmental stressors by enhancing tolerance to heat, cold, drought, and salinity (Whipps et al., 2008; Kembel et al., 2014; Martirosyan and Steinberger, 2014; Agler et al., 2016; Saleem et al., 2017). Several studies have suggested that the ecophysiological adaptation of desert plants to their harsh habitat is, at least partially, via microbial functional diversity (Redford et al., 2010; Martirosyan and Steinberger, 2014). While the exact correlation of phyllosphere microbial communities and these unique adaptations is yet to be clarified, a growing number of studies indicate that each plant species provides a unique microenvironment that is suitable for its specific bacterial communities (Camarena-Pozos et al., 2019; Flores-Núñez et al., 2019; Chaudhry et al., 2021).

Plant phyllosphere microbes were found to differ among different habitat and climate conditions when compared among arid, semiarid, and temperate habitats. For instance, Martirosyan et al. (2016) investigated the adaptation of three Negev desert plant species and found gram-negative Bacteroidetes to dominate the leaves of *Hammada scoparia* while practically absent in other neighboring desert plant species (Martirosyan et al., 2016). Plants desert microbiomes were also found to correlate with high temperature, droughts, and UV radiation (Qvit-Raz et al., 2008; Carvalho and Castillo, 2018), regardless of their geographical location (Finkel et al., 2012). In this context, desert phyllosphere microbes were shown to mediate plant growth and the metabolism of some nutrients by fixing nitrogen from atmospheric sources (Lambais et al., 2017), utilizing phosphorus through solubilizing enzymes (Mwajita et al., 2013; Batool et al., 2016) and producing siderophores to bind iron (Scavino and Pedraza, 2013; Fu et al., 2016), and even increasing plant resistance against pathogens such as *Botrytis* fungal infection (i.e., blight disease) (Li et al., 2012; Kefi et al., 2015).

Alongside the effects of seasonality (Redford and Fierer, 2009; Redford et al., 2010; Copeland et al., 2015) and canopy structure (Leff et al., 2015) on plant phyllosphere microbiome, other studies have shown that abiotic (climate-related) and biotic (plant genotype) factors also play an important role in structuring the phyllosphere microbial communities (Rastogi et al., 2013). Epiphytic (outside, on the surface the leaves) and endophytic (inside the leaf tissue) microbial communities were shown to be different in composition: epiphytic bacterial communities found to be more diverse and more abundant compared with the endophytic bacterial communities; moreover, abiotic factors were shown to have different effects on epiphytic and endophytic bacterial communities within the same plant host. The season was the major driver of community composition of epiphytes, whereas wind speed, rainfall, and temperature were the major drivers shaping endophytic composition (Gomes et al., 2018).

These complex interactions between the plant microbiome (both epiphytic and endophytic) and different biotic and abiotic conditions within arid ecosystems are of particular interest considering the current scenarios of climate change and desertification (Stringer et al., 2009). Additionally, studies on microbiomes in arid plants could shed new light on important key microbial groups that might be of potential use in arid agricultural practices, biotechnology, and plant adaptation strategies to climate change (Vacher et al., 2016).

**Figure 1:**
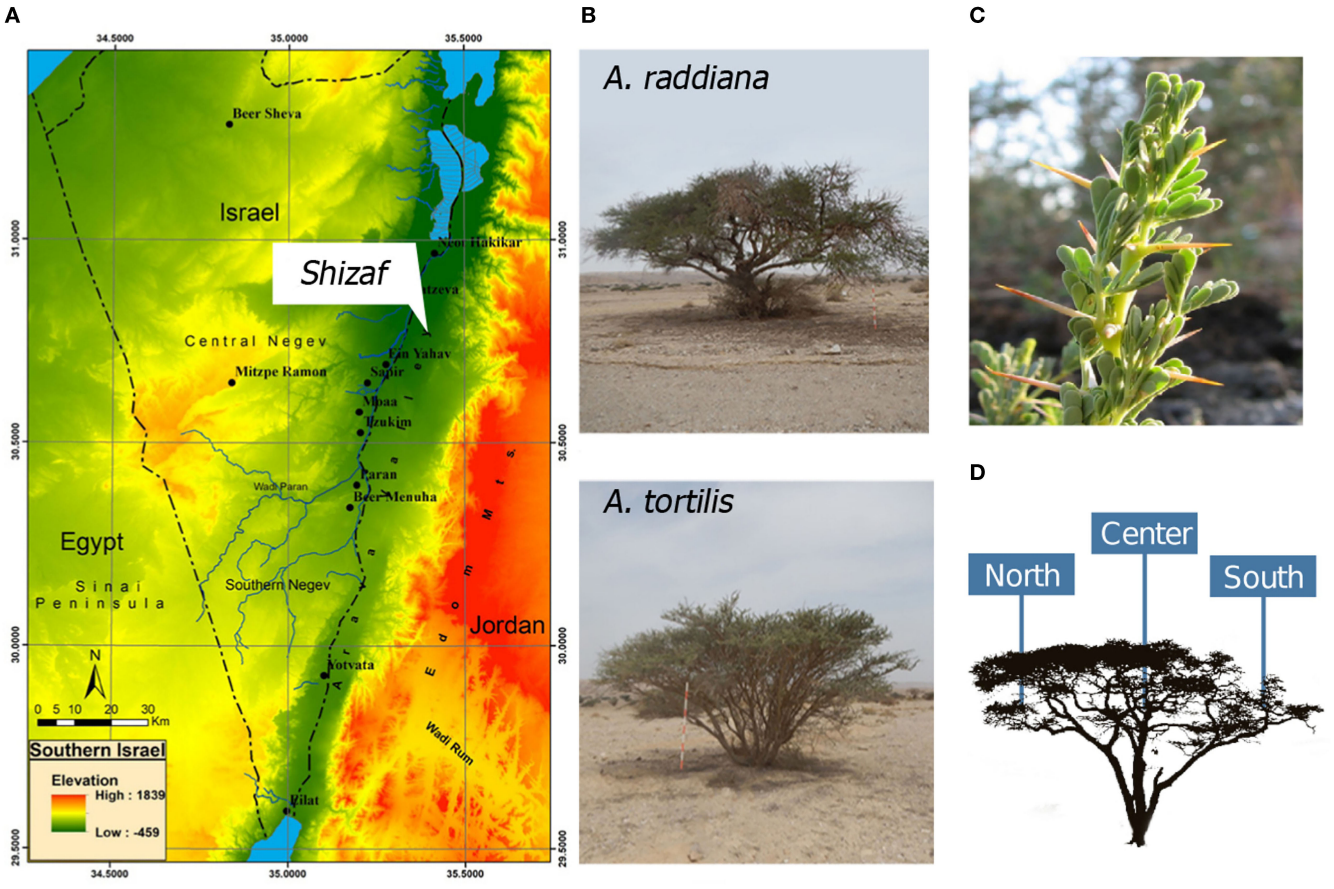
**(A)** Southern Israel topography map showing the study site, Wadi Shizaf, and **(B)** acacia trees (*A. raddiana* and *A. tortilis*) sampled monthly during 2015. In each month, **(C)** leaf samples were collected from the **(D)** north, center, and south sides of the canopies.

In this study, we investigated the epiphytic and endophytic microbiomes associated with the phyllosphere (leaves) of *Acacia raddiana* (Savi) and *Acacia tortilis* (Forssk) (Figure 1B) in the Arava Desert (Figure 1A). These two tree species grow in some of the hottest and driest places on Earth, such as the arid Arava Valley along the Dead Sea Transform (also known as Dead Sea Rift) in southern Israel and Jordan. In these arid habitats, *A. raddiana* and *A. tortilis* are the most abundant, and sometimes the only tree species present (Danin, 1983); they are mostly found growing in the channels of ephemeral river beds (Munzbergova and Ward, 2002). Both *A. raddiana* and *A. tortilis* are considered keystone species as they support the local plant and animal communities surrounding them and locally improve soil conditions for other plant species (Milton, 1995; Ward and Rohner, 1997; Munzbergova and Ward, 2002; Rodger et al., 2018; Winters et al., 2018). We hypothesized that variations in the bacterial communities of phyllosphere would be associated not only with the host species (*A. raddiana* and *A. tortilis*), but also with sampling season (temporal variations) and tree microclimate (leaves growing on the shade facing north side of the tree vs. leaves growing on the south side of the tree that are exposed to direct sun radiation; spatial variations) (Figure 1D).

## Materials and Methods

### Study Area and Sampling Scheme

This study was conducted in the Arava Valley, a hyperarid region along the Dead Sea Transform in southern Israel and Jordan. The elevation of the area ranges from 230 m above sea level to 419 m below sea level (Figure 1A). The climate in the Arava Valley is hot and dry: 30-year averages of minimum, mean, and maximum air temperatures of the hottest months were 26.2, 33.2, and 40.2°C, respectively; average minimum, mean, and maximum air temperature of the coldest months were recorded as 9.1, 14.4, and 19.6°C, respectively. Annual precipitation ranges between 20 and 70 mm/year and is restricted to the period between October and May (Winters et al., 2018) with large temporal (year-to-year) and spatial variations (Ginat et al., 2011). The combination of the very high air temperatures and the very low relative humidity values of 6% can cause summer midday vapor-pressure deficit (VPD) to reach 9 kPa (Winters et al., 2018). Vegetation in the region is usually confined to “Wadis” (ephemeral river beds; Ward, 2010), where the main water supply comes from underground aquifers (Sher et al., 2010; Winters et al., 2015) and winter flash floods (Shrestha et al., 2003). Multiple individual *A. raddiana* and *A. tortilis* trees are scattered throughout Wadi Shizaf (Figure 1A), but never form a continuous canopy. Wadi Shizaf is a dry sandy streambed at the northern edge of the Arava Valley, Israel (Figure 1A; 30°44′ N, 35°14′ E; elevation −137 m). Meteorological data (air temperature and humidity logged every 3 h) for this site were obtained from the Israeli Meteorological Service station 340528, located at Hatzeva only 7 km north of Wadi Shizaf (Supplementary Figure 1).

To sample bacteria from acacia trees in Wadi Shizaf, leaves from two neighboring *A. tortilis* trees (>20 m away from each other) (T023 and T300) and two neighboring *A. raddiana* trees (R284 and R286) were sampled monthly between January and December 2015 on their north, south, and central canopy sides (Figure 1D, Supplementary Table 1). This sampling scheme was chosen to enable us to investigate the effect of two different host (tree) species, in addition to the variation caused by the sampling season and the microclimate effect (different canopy sites—north, central, and south-facing sides of tree) on the phyllosphere microbiome.

During all sampling months, samples were collected from trees using sterile gloves (changed between each sample) between 9:00 and 11:00 a.m. Leaves (20–25 g fresh weight) were collected monthly (see Supplementary Table 1 for exact dates) and inserted into 15-mL sterile tubes placed on ice. Upon reaching the laboratory (within <2 h), samples were moved to freezers (−20°C) where they were kept until subjected to DNA extraction.

### DNA Extraction

All DNA extractions were performed using the MO BIO 96-well-plate PowerSoil DNA Isolation Kits (MO BIO Laboratories, Carlsbad, CA, USA). For epiphyte (the outer surface of the tree's leaves) microbial community extractions, 0.15 g (FW) of frozen leaves was weighed and placed in 1.5-mL Eppendorf tubes filled with 500 μL MO BIO Powerbead Solution and sonicated (DG-1300 Ultrasonic cleaner; MRC LAB, Israel) for 5 min. The solution was then transferred to the Powerbead Tubes, and the remaining steps for DNA extraction were carried out following the manufacturer's protocol. For the extraction of endophytic (inside the leaf tissue) microbial communities, leaf samples following epiphytic microbial extraction were washed using 1 mL of DNA/RNA-free water three times to eliminate the epiphyte microbiome fraction. Leaves were washed using 1 mL of DNA/RNA-free water three times to eliminate the epiphyte microbiome fraction. The washed leaves were then cut into small pieces using a sterile scalpel and placed into the MO BIO 96-well-Powerbead plate for DNA extraction following the manufacturer's protocol. All steps of DNA extraction were carried out in a sterile UV-hood (DNA/RNA UV-cleaner box, UVT-S-AR bioSan; Ornat, Israel) to reduce external contaminations. In every DNA extraction, using a 96-well-plate, DNA extraction negative controls were added by placing 200 μL of RNase-free water (Sigma–Aldrich, Israel). All samples were placed randomly in the DNA extraction plate to exclude any bias.

### Multiplex Polymerase Chain Reaction for Targeted Amplicon Sequencing of the 16S Ribosomal RNA Gene—Polymerase Chain Reaction I

To obtain a better phylogenetic resolution and diversity estimate, a multiplex polymerase chain reaction (PCR) using five different sets of the 16S rDNA genes was applied to cover approximately 1,000 bp of the 16S rRNA gene (Supplementary Table 2). First PCR (PCR-I) reactions were performed in triplicates, where each PCR-I reaction (total 25 μL) contained 12.5 μL of KAPA HiFi HotStart ReadyMix (Biosystems, Israel), 0.4 μL of equal vol/vol mixed primers forward and reverse primers (Supplementary Table 2), 10 μL of molecular graded DDW (Sigma, Israel), and 2 μL of (2–100 ng/μL) DNA template. PCR-I reactions were performed in a Biometra thermal cycler (Biometra, TGradient 48, Göttingen, Germany) with the following routine: initial denaturation 95°C for 2 min, followed by 35 cycles of 98°C for 10 s, 61°C for 15 s, and 72°C for 7 s. Ending the PCR-I routine was a final extension at 72°C for 1 min. Upon completion of PCR-I, an electrophoresis gel was run to verify that all the samples were amplified successfully. Following this quality control step, triplicate samples were pooled together and were cleaned using Agencourt® AMPure XP (Beckman Coulter, Inc, Indianapolis, IN, USA) bead solution based on manufacturer's protocol.

### Library Preparation and Next-Generation Sequencing

Library preparation began with performing a second PCR (PCR-II) in order to connect the Illumina linker, adapter, and unique 8-bp barcode for each sample (Fuks et al., 2018). The PCR-II reactions were prepared by mixing 21 μL of KAPA HiFi HotStart ReadyMix (Biosystems, Israel), 2 μL of mixed primers with the Illumina adapter (Supplementary Table 3), 12.6 μL of RNase-free water (Sigma, Israel), and 4 μL of each sample from the first PCR (PCR-I) product with 2 μL of barcoded reverse primer. These reactions were run in the thermal cycler with the following routine: initial denaturation at 98°C for 2 min, followed by, eight cycles of 98°C for 10 s, 64°C for 15 s, and 72°C for 25 s, these cycles were followed by final extension of 5 min at 72°C. Then, all PCR-II products were pooled together and subjected to cleaning using Agencourt® AMPure XP bead solution Beckman Coulter, Inc, Indianapolis, IN, USA) based on the manufacturer's protocol, where 50 μL of pooled PCR-II product was cleaned using 1:1 ratio with the bead solution for more conservative size exclusion of fragments <200 bp. As a final step, 50 μL of DDW with 10 mM Tris (pH 8.5) was added to each sample. This was followed by aliquoting 48 μL of the supernatant to sterile PCR tubes and storing in −80°C, while an additional 15 μL of the final product was sent to the Center for Genomic Technologies at the Hebrew University of Jerusalem (Jerusalem, Israel) where samples were sequenced on a full lane of 150-bp paired-end (to correct for sequencing error and enhance total read quality) reads using an Illumina MiSeq platform.

### Sequence Analysis and Quality Control

A series of sequence quality control (QC) steps were applied before data analysis. These included filtering PhiX contamination using Bowtie2 (Langmead and Salzberg, 2012), removing incomplete and low-quality sequences (phred Q threshold 33) by pairing the two reads using PEAR (Zhang et al., 2014), and identifying ambiguous bases and miss-merged sequences using mothur V.1.36.1 (Schloss et al., 2009). Following these QC steps, QIIME-1 (Caporaso et al., 2010) was used. Sequences were aligned, checked for chimeric sequences, and clustered to different operational taxonomic units (OTUs) based on 97% sequence similarity. Sequences were then classified based on the Greengenes database V13.8 (DeSantis et al., 2006), and an OTU table was generated. All sequences classified as f__mitochondria, c__Chloroplast, k__Archaea, and K__Unclassified were removed from the OTU table.

### OTU Richness and Diversity Estimates

For each sample, four diversity estimates were measured: (i) observed number of OTUs, (ii) Chao1 species' diversity estimate (Hill et al., 2003), (iii) Simpson diversity index (Keylock, 2005), and (iv) Shannon bacterial communities' diversity (Haegeman et al., 2013). All diversity metrics were calculated within QIIME-1 (Kuczynski et al., 2012) using the parallel_alpha_diversity.py command on the rarefaction subsamples set to 10,000 sequences using the multiple_rarefactions.py command.

### Assessment of Community Composition

From the obtained QIIME classified OTU table, each taxonomic group was allocated down to the genus level using the summarize_taxa.py command in QIIME and relative abundance was set as the number of sequences affiliated with that taxonomic level divided by the total number of sequences. Relative abundances were plotted using R statistical software (R Core Team, 2013), where each phylum was assigned a distinguished color, and all genera under the same phylum were assigned to different shades of the same color.

### Statistical Analysis

Using the VEGAN package (Oksanen et al., 2018) in R, nonparametric multidimensional scaling (NMDS) was used to produce ordination based on Bray–Curtis distance matrix based on the total sum transformed matrix for the raw OTU table (Sinclair et al., 2015). Canonical correspondence analysis (CCA) was used to plot abiotic factors and to show how they explain variance in the microbial communities (Gonzalez et al., 2008). Permutational multivariate analysis of variance (PERMANOVA) was used to test the effects of tree species, microclimate (different areas within the tree canopy), and seasonality and tree phenology on the epiphytic and endophytic microbiomes associated with these two trees using the adonis2 function in R (McArdle and Anderson, 2001); relative abundances of different bacterial phyla were tested using analysis of variance (ANOVA) with *post-hoc* Tukey honestly significant difference test for group significance.

## Results

A total of 139 acacia leaf samples [two tree species × two replicate trees from each species × 3 canopy locations for epiphytes (but only the south canopy for endophytes) × 9 months] were collected for both epiphytic and endophytic microbial communities (Supplementary Table 1; notice that a total of seven samples were lost during sample processing before sequencing) and sequenced for their 16S rRNA genes using our five different primer sets (Supplementary Table 2). The average sequence number per each primer set varied significantly for the different regions of amplification (Supplementary Table 2). Results showed that the third primer set (F649 and R889) obtained the highest number of raw sequences with an average raw sequence number of 38,683 ± 18,723; thus, we based all further analysis on the F649-R889 primer set. This primer set retained its rank among all other primer sets even after the QC procedure, with 15,424 ± 13,784 high-quality sequences.

### Bacterial Community Composition of Epiphytic vs. Endophytic Assemblages

For comparing epiphytic and endophytic bacterial community structures, we used the leaf samples that were collected only at the south (“S”) canopy side of the acacia trees (Supplementary Table 1). Diversity estimates, including observed number of OTUs, Chao1, Simpson diversity index, and Shannon–Wiener, were calculated for both *A. raddiana* and *A. tortilis* (Table 1). Epiphytic bacterial diversity was higher when compared to endophytic bacterial communities (Table 1), indicating a different bacterial structure in the external vs. internal parts of the leaves.

**Table 1:** Average diversity estimates (±SD) of microbial communities of *A. raddiana* and *A. tortilis* measured across the entire sampling months for the epiphyte at north (N) and center (C) canopy sides and for both epiphytes and endophytes at south (S) canopy side.

**Species**	**Canopy**	**Observed no. of OTUs**	**Chao1**	**Simpson diversity index**	**Shannon–Wiener diversity index**
*A. raddiana*	N-epiphyte	460.5 ± 273.1	716.8 ± 367.6	0.8 ± 0.2	3.5 ± 1.1
	C-epiphyte	387.3 ± 228.1	614.7 ± 327.1	0.9 ± 0.1	3.4 ± 1.0
	S-epiphyte	445.5 ± 210.2	680.9 ± 300.3	0.9 ± 0.1	3.6 ± 0.8
	S-endophytes	236.5 ± 42.6	368.5 ± 89.4	0.6 ± 0.2	1.9 ± 0.7
*A. tortilis*	N-epiphyte	607.8 ± 290.7	902.7 ± 387.5	0.9 ± 0.1	3.8 ± 0.8
	C-epiphyte	519.7 ± 254.4	811.0 ± 373.2	0.8 ± 0.2	3.3 ± 1.0
	S-epiphyte	512.5 ± 262.7	754.2 ± 349.6	0.9 ± 0.1	3.4 ± 0.7
	S-endophytes	148.3 ± 46.9	242.7 ± 80.8	0.6 ± 0.2	1.8 ± 0.6

**Figure 2:**
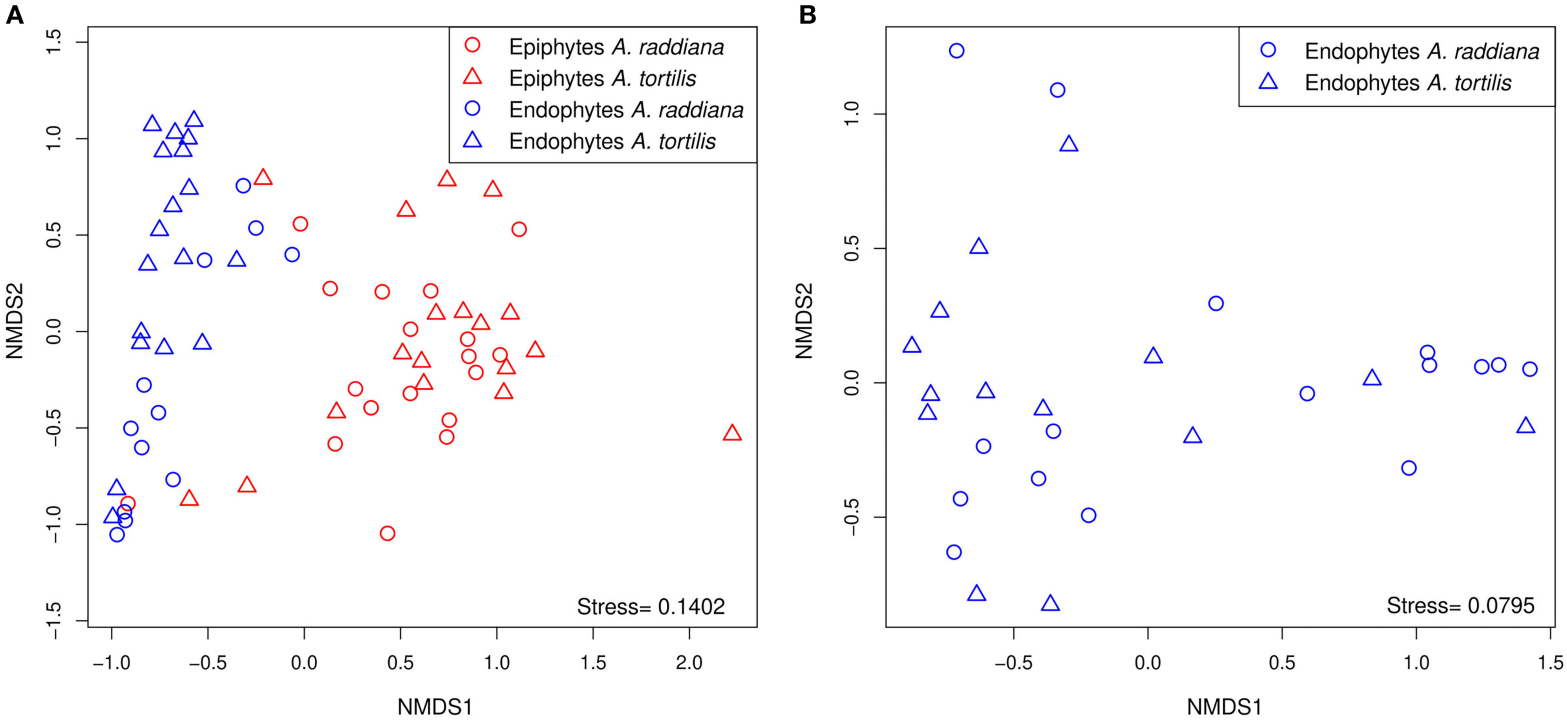
NMDS illustrating the phyllosphere bacterial community; separate clusters of bacterial communities are evident for **(A)** the epiphytic (red) and the endophytic (blue) bacterial communities from leaves sampled from south side canopy areas; and **(B)** unique clusters of endophytic bacterial communities observed in *A. raddiana* (blue) and *A. tortilis* leaves.

**Figure 3:**
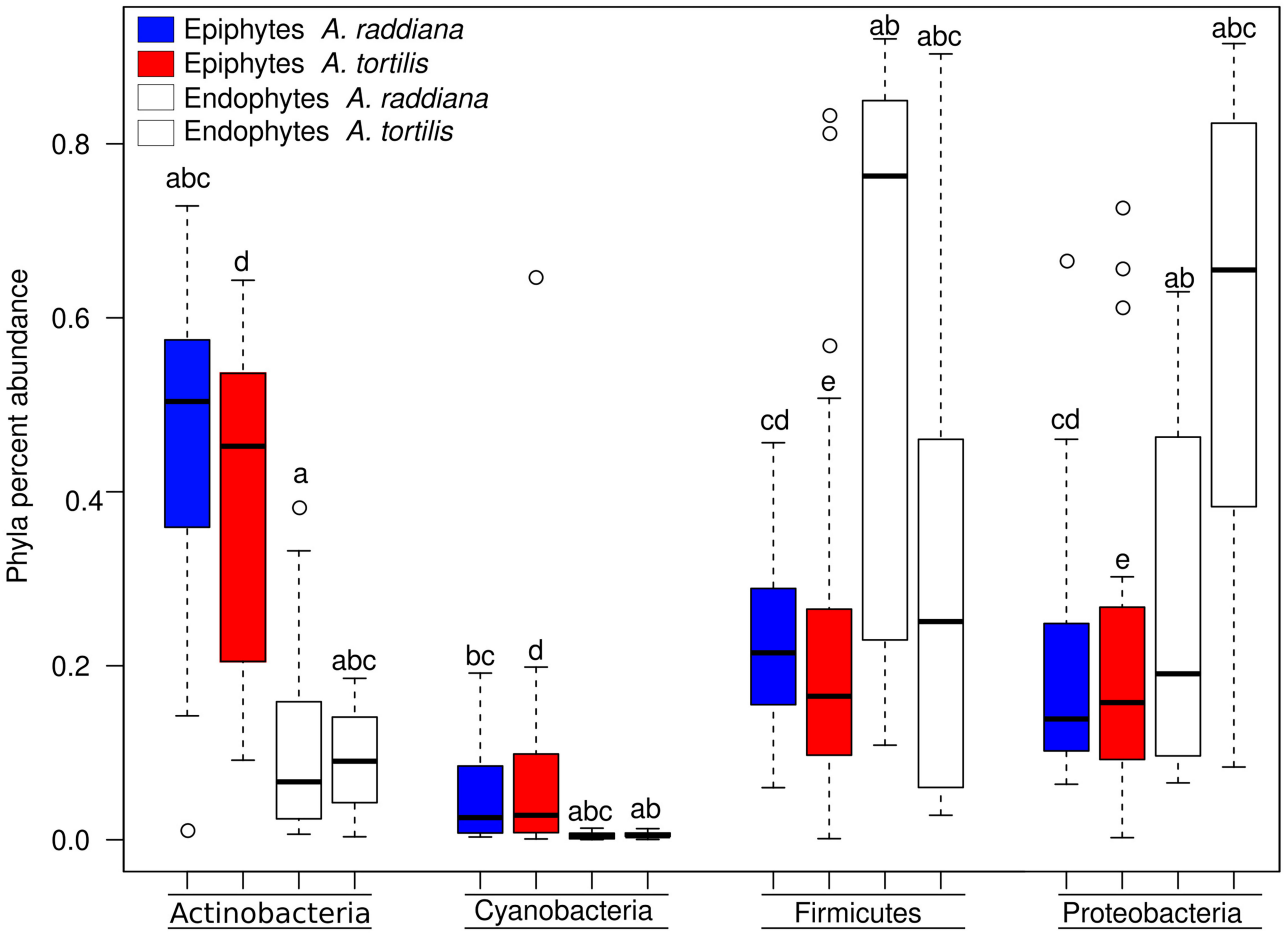
Box plot illustrating the percent abundance of epiphyte and endophyte major bacterial phyla.

To compare the diversities of epiphytic and endophytic bacterial communities extracted from leaf samples, acacia samples from south-facing canopies were analyzed and plotted using NMDS, based on the Bray–Curtis distance matrix (Figure 2). The NMDS showed two separate clusters of epiphytic and endophytic bacterial communities, statistical analysis Using PERMANOVA found endophytic and epiphytic microbial communities to be significantly different (*p* = 0.001; Figure 2A). While the epiphytic bacterial communities from the two acacia species (*A. raddiana* and *A. tortilis*) did not demonstrate separate clusters (*p* = 0.134, Table 2, Figure 2A), the endophytic bacterial communities were found to be significantly different for both acacia species (*p* = 0.021, Table 3, Figure 2B). To illustrate these differences, we plotted the bacterial phylum with more than 5% of the total community composition (Figure 3) and performed Tukey test of significance on the log-transformed abundances to normalize the variance. Results showed a higher median abundance of Actinobacteria in *A. raddiana* and *A. tortilis*, when comparing epiphytic to endophytic bacterial communities. However, these differences were only significant for *A. tortilis* (45.2 ± 17.7% and 9.0% ± 5.9%, *p* < 0.05) and not for *A. raddiana* (44.5 ± 19.2% and 5.7 ± 10.0%, *p* > 0.05; Figure 3). Similarly, Cyanobacteria median abundance was higher in epiphytic compared to endophytic bacterial communities and was significant for *A. tortilis* (2.6% ± 5.8% and 0.4 ± 0.4%, *p* < 0.05) and not for *A. raddiana* (2.5 ± 5.2% and 0.5 ± 0.4%, *p* > 0.05; Figure 3). On the other hand, the abundance of both Firmicutes and Proteobacteria was lower in epiphytic compared to endophytic bacterial communities and was significantly different among the two acacia species (*p* < 0.05). The median abundance of Firmicutes in *A. raddiana* epiphytes was 21.4 ± 10.1% compared to 76.3 ± 32.4% in endophytes and in *A. tortilis* was 15.8 ± 12.2% and 25.1 ± 27.6%, respectively (Figure 3). The median abundance of Proteobacteria in epiphytes and endophytes of *A. raddiana* was 13.5 ± 12.1% and 19.1 ± 21.3%, and that of *A. tortilis* was 13.2 ± 8.4% and 65.5 ± 26.2%, respectively (Figure 3).

**Table 2:** PERMANOVA analysis showing statistical significance of epiphytic microbial communities across sampling months, season, tree phenology (leaves shedding period), and canopy sides.

	***Df***	**SumOfSqs**	***F*. Model**	***R^**2**^***	**Pr(>F)**
Season	3	2.5079	4.3842	0.10296	0.001
Month	5	3.3865	3.5520	0.13903	0.001
Shedding	1	0.1722	0.9031	0.00707	0.552
Acacia_species	1	0.2570	1.3479	0.01055	0.134
Month:Acacia_species	7	1.5159	1.1357	0.06223	0.139
Acacia_species:Tree	2	0.4866	1.2759	0.01998	0.114
Acacia_species:Canopy	4	0.7775	1.0194	0.03192	0.410
Residuals	80	15.2542		0.62626	
Total	103	24.3578		1.00000	

*df, degrees of freedom; SumOfSqs, sum of squares*.

**Table 3:** PERMANOVA analysis showing statistical significance of epiphytic and endophytic microbial communities across sampling months.

	***Df***	**SumOfSqs**	**F. Model**	***R^**2**^***	**Pr(>F)**
Shedding	1	0.5609	3.9882	0.10529	0.029
Acacia_species	1	0.6189	4.4006	0.11629	0.021
Season	3	0.4261	1.0100	0.08007	0.418
Month	5	1.0376	1.4756	0.19497	0.206
Acacia_species:Tree	2	0.1807	0.6425	0.03396	0.594
Acacia_species:Season	3	0.5998	1.4217	0.11271	0.219
Acacia_species:Month	2	0.2101	0.7470	0.03948	0.513
Residuals	12	1.6876		0.31712	
Total	29	5.3217		1.00000	

*df, degrees of freedom; SumOfSqs, sum of squares*.

### Temporal, Seasonality, Phenology, and Spatial Variation (Canopy Side) of Phyllosphere Bacterial Communities

**Figure 4:**
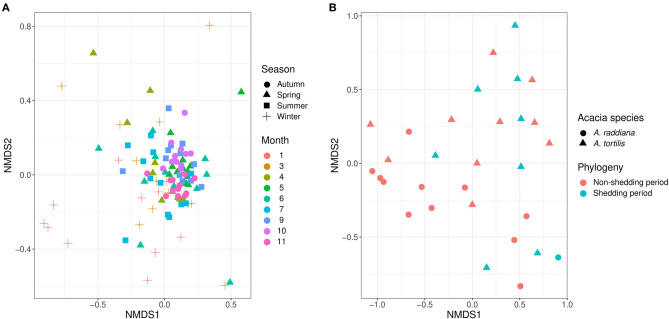
NMDS illustrates **(A)** the epiphytic phyllosphere bacterial communities at different sampling seasons and moths and **(B)** endophytic for different acacia species and tree phenology.

To test the temporal effect (sampling month), seasonality, tree phylogeny (leaves shedding time), and canopy variation on the epiphytic bacterial communities of both *A. raddiana* and *A. tortilis*, we have performed PERMANOVA analysis of independent variables and nested models (Table 2). Table 2 shows both seasonality (winter, spring, summer, and autumn) and different sampling month had a significant effect of the epiphytic microbial communities (*p* = 0.001), whereas the effect of leaves shedding period (tree phenology), Acacia species (*A. raddiana* and *A. tortilis*), Tree (2 individual tree for each acacia species), and canopy side (north, center, and south) had no significant effect of the epiphytic bacterial diversity (*p* > 0.05). To better illustrate the microbial communities at different sampling months and seasonality, we plotted NMDS (Figure 4A) and dispersion (variance) to the centroid for the different months and seasons (Supplementary Figure 4), showing different clusters based on sampling month and season. We have also plotted bar plots illustrating the bacterial families composition for OTU > 1% at different canopy sides over the sampling months showing bacterial families to not significantly change between the different canopy sides, whereas main changes in the different sampling months were related to higher abundance of *Bacillaceae* in January, *Enterobacteriaceae* in March and April, and *Xanthomonadaceae in* May, June, and July (Supplementary Figure 5).

For endophytic bacterial communities, we have also checked the effect of sampling months, seasonality, tree phylogeny, and acacia species on endophytic bacterial diversity using PERMANOVA analysis (Table 3). Table 3 shows tree phenology (leaves shedding period) and acacia species (*A. raddiana* and *A. tortilis*) have a significant effect on the endophytic bacterial diversity (*p* = 0.029 and 0.021, respectively), whereas the effect of different sampling seasons, months, individual trees within each species had no significant effect (*p* > 0.05). To better illustrate the significant effect of tree phylogeny (shedding period) and acacia species, we plotted NMDS (Figure 4B) and dispersion (variance) to the centroid (Supplementary Figure 6) to show the different clusters following tree phenology and acacia species. When bar plots were plotted illustrating the bacterial families composition for ASV > 1% at different phylogeny (shedding period vs. non-shedding period) and for different acacia species (Supplementary Figure 7), higher abundance of *Bacillaceae* was observed during non-shedding period “new,” whereas *Comamonadaceae* showed a higher abundance at leaves shedding period relative to each other (Supplementary Figure 7A). Similarly, higher abundance of *Bacillaceae* relative to *Comamonadaceae* was observed for *A. raddiana* compared to *A. tortilis* (Supplementary Figure 7B).

### Effect of Changes in Abiotic Factors on Variation in the Microbial Communities

**Figure 5:**
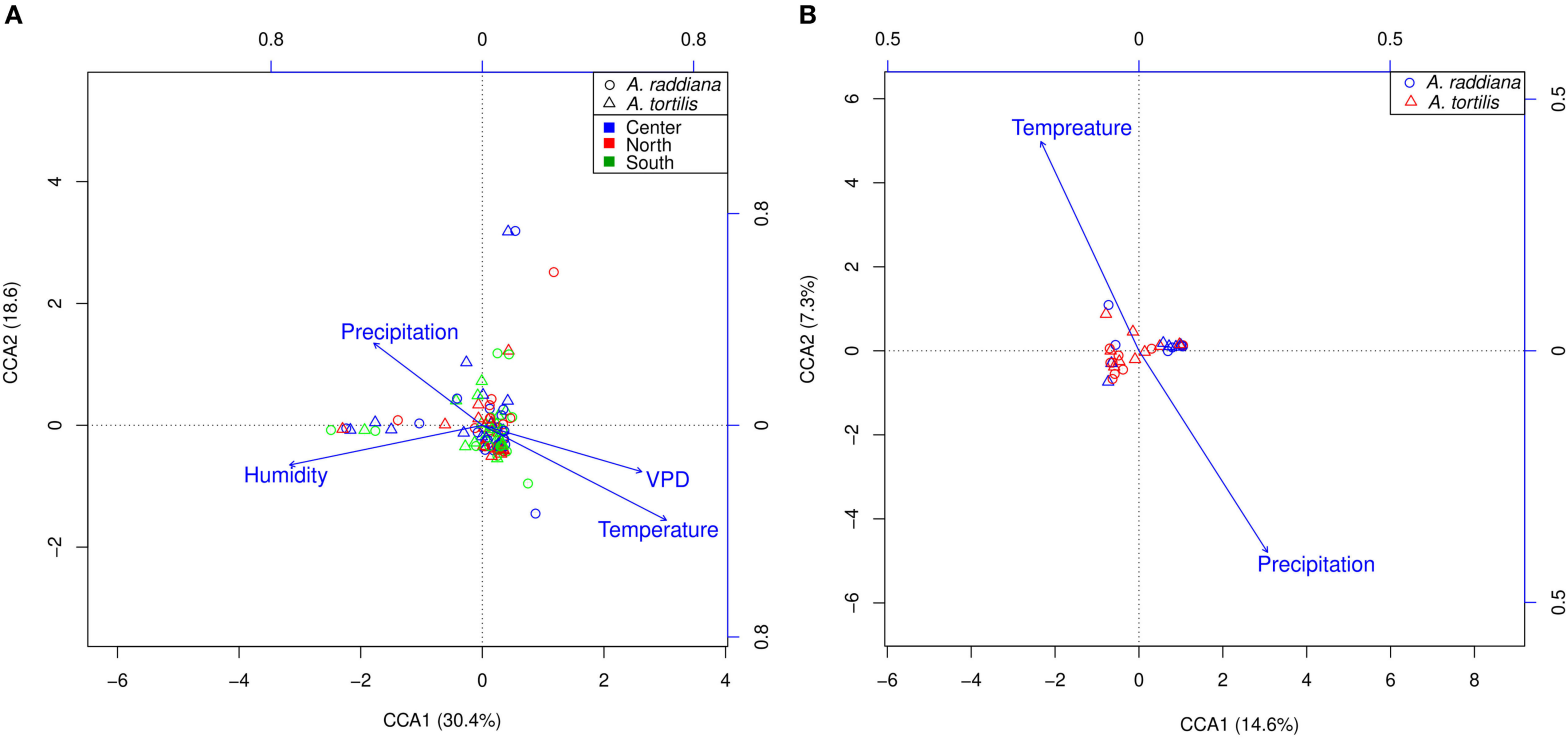
CCA ordination illustrating **(A)** epiphytic bacterial community at north (red), south (green), and center (blue) canopy sides and **(B)** endophytic bacterial communities, for *A. raddiana* (blue circles) and *A. tortilis* (red triangles) with significant abiotic factors affecting the bacterial communities.

To test abiotic factors' effect on the microbial communities, CCA (ter Braak, 1986) was performed for the epiphytic (Figure 5A) and endophytic (Figure 5B) bacterial communities of *A. raddiana* and *A. tortilis*. Only those abiotic factors with significant values (*p* ≤ 0.05) were plotted. Results show that air temperature, VPD, humidity, and precipitation had a significant effect on the variability of epiphytic bacterial communities and were able to explain up to 49% (30.4 and 18.6% on CCA axes 1 and 2, respectively) of total epiphytic community variability (Figure 5A). In comparison, temperature and precipitation (but not VPD) had a significant but slightly weaker effect on the endophytic bacterial communities, explaining up to 23.9% (16.6 and 7.3% on CCA axes 1 and 2, respectively) (Figure 5B) of total endophytic community variability.

### Bacterial Family Abundances

**Figure 6:**
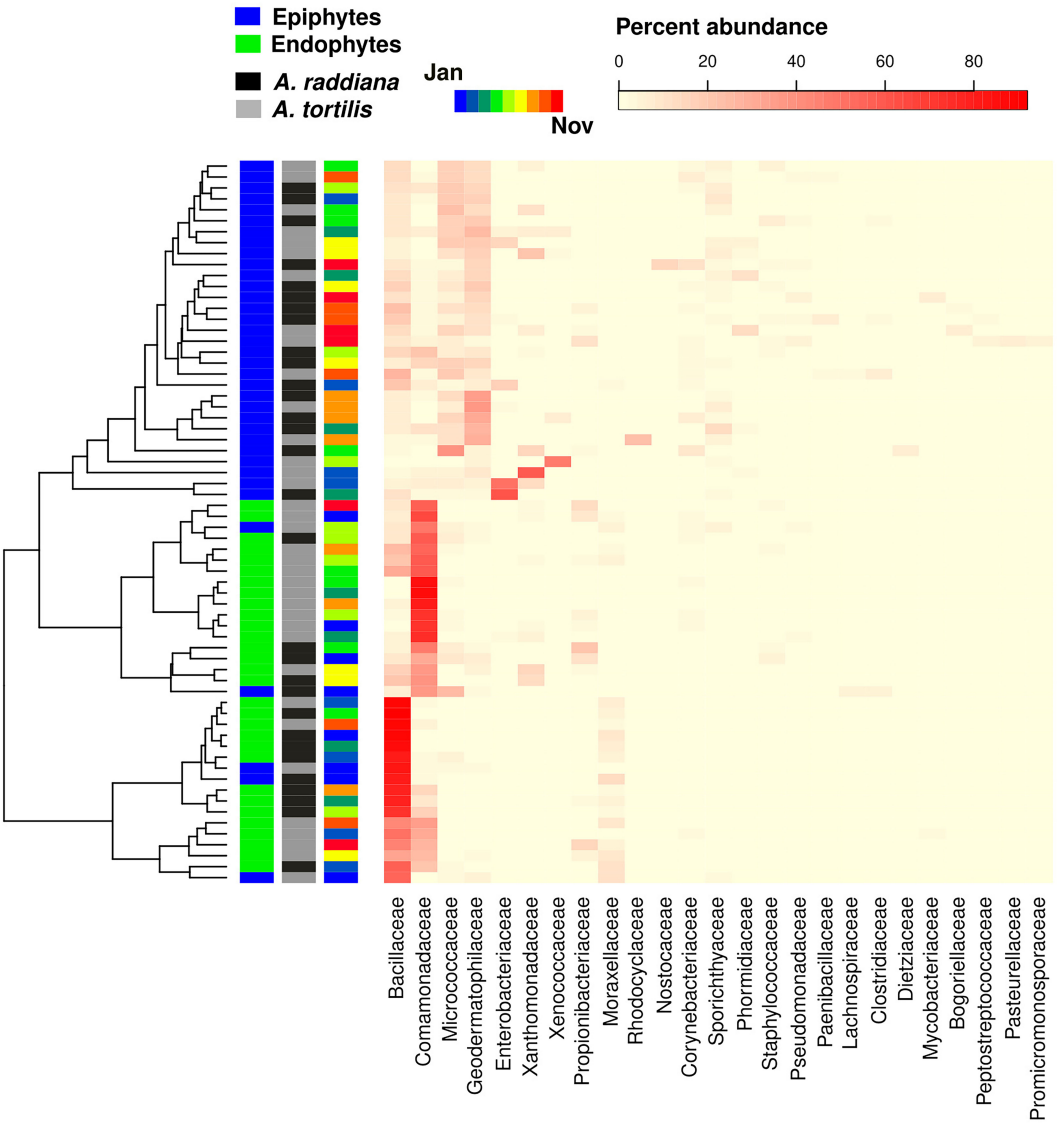
Heatmap showing the abundance of bacterial family abundances (*x* axis) for each of the sampled epiphytic and endophytic bacterial communities at the south canopy side at different sampling months (January–November).

To test for the major changes in bacterial family abundances, a heatmap was made to show epiphytic and endophytic bacterial diversities at the family level (Figure 6). Results show that only a few bacterial OTUs were differentially abundant comparing epiphyte and endophytes, or when comparing within the endophytic communities between *A. raddiana* and *A. tortilis*. Epiphytic bacterial communities were mainly dominated by *Geodermatophilaceae, Micrococcaceae, Comamonadaceae*, and *Bacillaceae* bacterial families, whereas endophytic bacterial communities were dominated only by alternating abundances of the *Bacillaceae, Comamonadaceae*, and *Moraxellaceae* families (Figure 6, Supplementary Figure 8). In the endophytic bacterial communities, these changes in abundance correlated with different months of the year (Figure 6, Supplementary Figure 5).

## Discussion

To improve our understanding of the microbial structure of the phyllosphere microbiome of plants growing in extreme arid environments, we applied a high temporal and spatial resolution sampling scheme in two desert keystone trees (*A. raddiana* and *A. tortilis*). We investigated both the epiphytic and endophytic bacterial communities to understand the (i) intraindividual and interindividual spatial variation of the microbial communities within a tree—the spatial variation within the same tree caused by sun exposure (only for epiphytic microbial communities) and the variation between neighboring trees of the same species sampled at the same time and site (ii) variation of the microbial community caused by the host (tree) species (i.e., *A. raddiana* compared with neighboring *A. tortilis*), (iii) temporal variation of the microbial communities within the same tree species, canopy side, and individual trees sampled during different seasons.

Our results demonstrate that the epiphytic bacterial communities were more sensitive to changes in environmental abiotic conditions, compared with the endophytic bacterial communities that were more stable between different environmental conditions (e.g., seasons) but varied among host tree species. Surprisingly, up to 60% of the total bacterial communities (the combined epiphytic and endophytic microbiome populations) were unclassified below family level, highlighting the uniqueness of the microbiome associated with acacia trees in the arid environment of the Arava. When actinobacterial differences were compared in tree grove, shrub, meadow, desert, and farm soil ecosystems, the majority of unclassified actinobacterial sequences were found in desert ecosystems, accounting for ~50% of total Actinobacteria phylum (Zhang et al., 2019). Similar results from a study of desert soil in Pakistan indicate that a bulk portion of the OTUs were assigned to unclassified taxa (Amin et al., 2020).

In terms of the overall observed number of OTUs, Chao1, Simpson, and Shannon–Wiener diversity indices, the diversity of the epiphytic bacterial community was shown to be double that of its endophytic bacterial community counterpart (Table 1). While the average number of classified bacteria sequences for epiphytes was slightly higher (16,752) compared to endophytic (14,857) bacterial communities, the sequence number in each sample had no effect on the obtained diversity indices (Supplementary Figure 2). The higher abundance and richer microbial communities in epiphytes compared to endophytes were also observed in young and mature leaves of *Origanum vulgare*, where the total number of colony-forming units (CFUs) of epiphytic bacterial communities (5.0 ± 0.2) was more than double the CFUs of the associated endophytic communities (1.8 ± 0.1) (Pontonio et al., 2018). However, our results contradict previous work on microbiomes associated with *Arabidopsis thaliana* where epiphytic bacterial diversity indices were found to be lower than those measured for the associated endophytic bacterial communities (Bodenhausen et al., 2013). Like the work shown here, a recent study on the epiphytic and endophytic fungal diversity in leaves of olive trees (*Olea europaea*) growing in Mediterranean environments also showed that the epiphytic fungal communities had higher diversity indices compared to the endophytic diversity estimates (Gomes et al., 2018); similarly, bacterial endophytic diversity was lower compared to epiphytic diversity in tomato plants (Dong et al., 2019). The fact that our epiphytic OTU diversity was higher than the endophytic diversity is particularly surprising, considering previous work by Thapa and Prasanna (2018) that suggested that the conditions inside the plant might be more favorable compared with the more hostile conditions on the outside (Thapa and Prasanna, 2018). This might explain finding where diversity was higher for endophytic microbiomes (Bodenhausen et al., 2013). In our case, however, both *A. raddiana* and *A. tortilis* had a lower endophytic bacterial diversity compared to epiphytic bacterial diversity throughout the sampling period (Supplementary Table 4); the lower diversity of endophytic microbial communities compared to epiphytic communities may be a result of plant stress and physiological conditions regulated by stomatal opening (Arndt et al., 2004; Chaudhry et al., 2021). In fact, Chao1 richness in epiphytic microbial communities in *A. raddiana* and *A. tortilis* was higher in January, March, and November compared to summer months and corresponded to plant water VPD, indicating the importance of the physiological state of the plant in shaping endophytic bacterial communities (Supplementary Figure 3, Supplementary Table 4). These plant responses were shown to reduce the entry of epiphytes to the endosphere, thus affecting the plant's microbiome colonization (Pontonio et al., 2018; Remus-Emsermann and Schlechter, 2018; Schlechter et al., 2019).

Our results demonstrate that the epiphytic and endophytic bacterial communities are significantly unique (Figures 2A, 3, Supplementary Figure 8). We also found that the endophytic (but not the epiphytic) bacteria communities differed between the two acacia species (Figures 2A,B, Tables 2, 3, Supplementary Figures 6B, 8), with each host being associated with specific endophytic communities. In fact, many studies have indicated that the composition and abundance of endophytes in plants are synergistically determined by plant genotype and environmental factors (Terhonen et al., 2019). Plant tissue characteristics highly affect microbial abundance; thus, endophyte enrichment varies widely in different tissues within a plant (Baldrian, 2017). The significant effect of genotype on composition of endophytic microbial communities has been documented (Bodenhausen et al., 2014; Hardoim et al., 2015; Müller et al., 2015), and it is more severely affected by genotypic factors than by abiotic factors (Whipps et al., 2008; Rastogi et al., 2013; Agler et al., 2016). On the other hand, tree phenology may play an important role in phyllosphere microbiome; recent work by Winters et al. (2018) followed the dynamics in stem diameter, leaf phenology, and sap flow of *A. raddiana* and *A. tortilis* trees growing at the same sheizaf site as the trees presented here, during 3 consecutive years. While it was expected that stem growth and other tree activities would be synchronized with and limited to single rainfall or flash flood events that occur in the winter, Winters et al. (2018) found that cambial growth of both Acacia species actually stopped during the wet season and occurred during most of the dry season, coinciding with maximum daily temperatures as high as 45°C and VPD of up to 9 kPa. Summer growth was correlated with peak sap flow in June, indicating that trees relied year-round on deep soil water (Winters et al., 2018). Particularly relevant to the microbiome results presented here were the phenology changes demonstrated by the 3-year study by Winters et al. (2018) that showed that in the two acacia species, new leaves emerged twice a year, in early March and again in late October. The leaf-shedding period was relatively short (July–August) for *A. raddiana* and slightly earlier and longer (May–September) for *A. tortilis*. In this study, we applied those findings on our data, and there seems to be a strong link between the seasonal leaf phenology and the monthly diversity indices (Supplementary Table 4). The highest diversity was seen in older leaves, leaves that have been on the tree the longest time (i.e., June and July, Supplementary Figure 3). When we statistically checked the effect of tree phylogeny in both epiphytic and endophytic microbial community, we found endophytes but not epiphytes to be affected by tree phenology (leaves shedding period) (Tables 2, 3; Figure 4B, Supplementary Figures 6A, 7A).

Like other findings indicating the changes in bacterial communities in the phyllosphere due to different environmental and biotic factors (Remus-Emsermann and Schlechter, 2018; Schlechter et al., 2019), our results show that seasonality (temperature and month) is the major driver of community composition in epiphytic bacteria (Figures 4A, 5A; Table 2). Humidity, temperature, precipitation, and VPD were shown to have a strong effect explaining 30.4 and 18.6% in CCA axes 1 and 2, respectively, accounting for the total variance in microbial community composition (Figure 5A). Studies found that leaves' microbial communities can be significantly affected by leaves' moisture (Beattie, 2002; Lindow, 2006), bacteria also found to produce surfactants to increase the wettability of the leaf and lessen the ability of the cuticle to limit water accumulation (Schreiber et al., 2005). Although the effect of moisture from dew on the leaf community has not been explored, it is expedited to act similarly to rain (Stone et al., 2018).

In the endophytic bacterial communities, temperature and precipitation explained 14.6 and 7.3%, respectively, of the total microbial community (Figure 5B). It was suggested that abiotic regulatory factors affect the physicochemical properties of the leaves; in addition, these abiotic factors can affect external biotic factors (e.g., insects and pathogens), which in turn affect the plant's immunity and biology and therefore affect plant-associated microbiome (Liu et al., 2020). Nonetheless, plants undergo remarkable physiological changes in relation to abiotic factors; such changes can affect the availability of nutrients, water, and a wide range of secondary metabolites on the leaf surface and therefore significantly affect the epiphytic microbial communities (Liu et al., 2020). These findings can also be related to dust accumulation on leaves; dust is considered an important source of nutrients and essential metals in arid ecosystems (Reynolds et al., 2001, 2006). Aeolian dust also emerged as a significant vehicle for long-range transport of microorganisms (Maki et al., 2019), affecting plant microbiomes (Brown and Hovmøller, 2002; Banchi et al., 2019). Although in this study dust samples were not collected, in summer 2018 we have collected Aeolian dust in a pocket nearby the current study's sampled acacia trees; we also collected samples for endophytes and epiphytes from both *A. raddiana* and *A. tortilis*. The dust microbiome showed to cluster separately and closer to epiphyte bacterial communities (Supplementary Figure 9). The results also showed that dust samples are composed of two main bacterial families *Pseudomonadaceae* and *Halomonadaceae*, which were also dominant in the epiphytic microbial communities of both *A. raddiana* and *tortilis*; nonetheless, acacia epiphytes also showed a significant abundance of other bacterial families that were not presented in the collected dust samples (Supplementary Figure 9). These results suggest that desert dust microbiomes also can play an important role in desert epiphytes.

The effect of microclimate (i.e., the spatial variation caused by the different canopy sides) on the epiphytic bacterial diversity (Tables 1, 2) and community composition (Supplementary Figure 5) was shown to be significant. However, we only tested the effect of exposure to irradiance on epiphytes. A recent study investigated the endophytes of roots and leaves of *Oxyria digyna* showing the strong impact of tissue type on the endophytic bacterial community structure (Given et al., 2020). Assessing the different canopy sides showed that exposure to the sun significantly affects the physiological state of leaves (Hussain et al., 2020) and forms distinct epiphytic microbial communities; therefore, the effect of the canopy side on the endophytic phyllosphere microbial communities needs further investigation.

Our microbial community compositions also differ from those found in the phyllosphere of plants from subtropical and temperate regions, which are mostly dominated by Alphaproteobacteria (72%) (Laforest-Lapointe et al., 2016), Bacteroidetes (8%), and Acidobacteria (17%) (Kim et al., 2012), whereas ours were dominated by Firmicutes, Proteobacteria (mainly Betaproteobacteria, Figure 6), and Actinobacteria (Figure 3), shown to agree with other findings in arid system– and desert-adapted plant species (Citlali et al., 2018). The major differences between epiphytic and endophytic bacterial communities were due to the differential abundance of four major unclassified OTUs belonging the bacterial families of *Bacillaceae* (Firmicutes phylum) and *Comamonadaceae* (Betaproteobacteria phylum) for the endophyte of *A. raddiana* and *A. tortilis*, respectively (Figure 6). Other unclassified OTUs belonging to the bacterial families of *Geodematophilaceae* and *Micrococcaceae* (both belonging to Actinobacteria phylum) were found in the epiphyte bacterial communities (Figure 6). These bacterial families were also found in other extreme-condition studies that investigated the metagenomic signatures of the phyllosphere of *Tamarix aphylla* (Finkel et al., 2011, 2012, 2016) and other desert shrubs (Martirosyan et al., 2016), highlighting the relationships between these bacterial communities and their importance in the adaptation of desert plants to arid environments. However, the exact link between these different bacterial groups and their functional diversity is still to be investigated; such studies could shed light on the specific metabolites and enzymes that these adaptive bacterial groups exhibit in arid environments. With the growing interest in manipulating and inoculating food crops with particular microbial communities to extend shelf life and improve plant resilience and product taste, the long-coevolved microbiome of desert plants might have biotechnological potential. Desert plant microbiome may enhance the resilience of crop plants during ongoing processes of desertification and soil salinization expected to affect vast regions of the world in coming decades.

## Conclusions

The evolutionary relationships and interactions between plants and their microbiome are important for their adaptation to extreme conditions. In this study and based on 16S rDNA sequencing, we explored the spatiotemporal relationships between naturally occurring desert plants and their microbiome. While changes in the plant microbiome can affect plant development, growth, and health, we presented the effects of plant physiological conditions, temporal changes, canopy structure, abiotic parameters, and plant genotype and phenology on both epiphytic and endophytic bacterial communities of desert plant phyllosphere. Moreover, we showed how the desert plant phyllosphere is inhabited by distinct microbial communities compared to temperate and humid regions, stressing that a large portion of these microbial communities is not classified below family level. Our results shed light on the specific bacterial families and diversity patterns in relation to desert phyllosphere epiphyte and endophytes associated with extreme environmental conditions. The agritech (agritechnology) potential of these unique microbial communities calls for more research on the functionality of these epiphytic and endophytic microbial communities.

## Data Availability Statement

The original contributions presented in the study are publicly available. This data can be found here: All curated sequences were joined into a single fasta file and submitted to MG-RAST under project link (https://www.mg-rast.org/linkin.cgi?project=mgp92155). All codes for quaration steps, quality control and sequence analysis uploaded to GitHub repository, including the metadata files and made publicly available (https://github.com/ashrafashhab/Desert-plant-microbiome).

## Author Contributions

AA was involved in the project conceptualization, data curation, formal analysis, methodology, project administration, resources, visualization, and MS writing. SM, MB, and GW were involved in funding acquisition, project conceptualization, and project investigation. In addition, both GW and YB-L took an active part in MS and graphics revision and editing. RA-S and HD helped in laboratory and field work. All authors contributed to the article and approved the submitted version.

## Conflict of Interest

The authors declare that the research was conducted in the absence of any commercial or financial relationships that could be construed as a potential conflict of interest.
