# Supplementary material for: Temporal and Spatial Changes in Phyllosphere Microbiome of Acacia Trees Growing in Arid Environments
Source: Front Microbiol. 2021 Jul 12;12:656269. doi: 10.3389/fmicb.2021.656269 (PMC8312645; doi:10.3389/fmicb.2021.656269)
Supplement: Supplementary file 1 [file Data_Sheet_1.docx]

***Supplementary Material***

1. **Supplementary Figures**


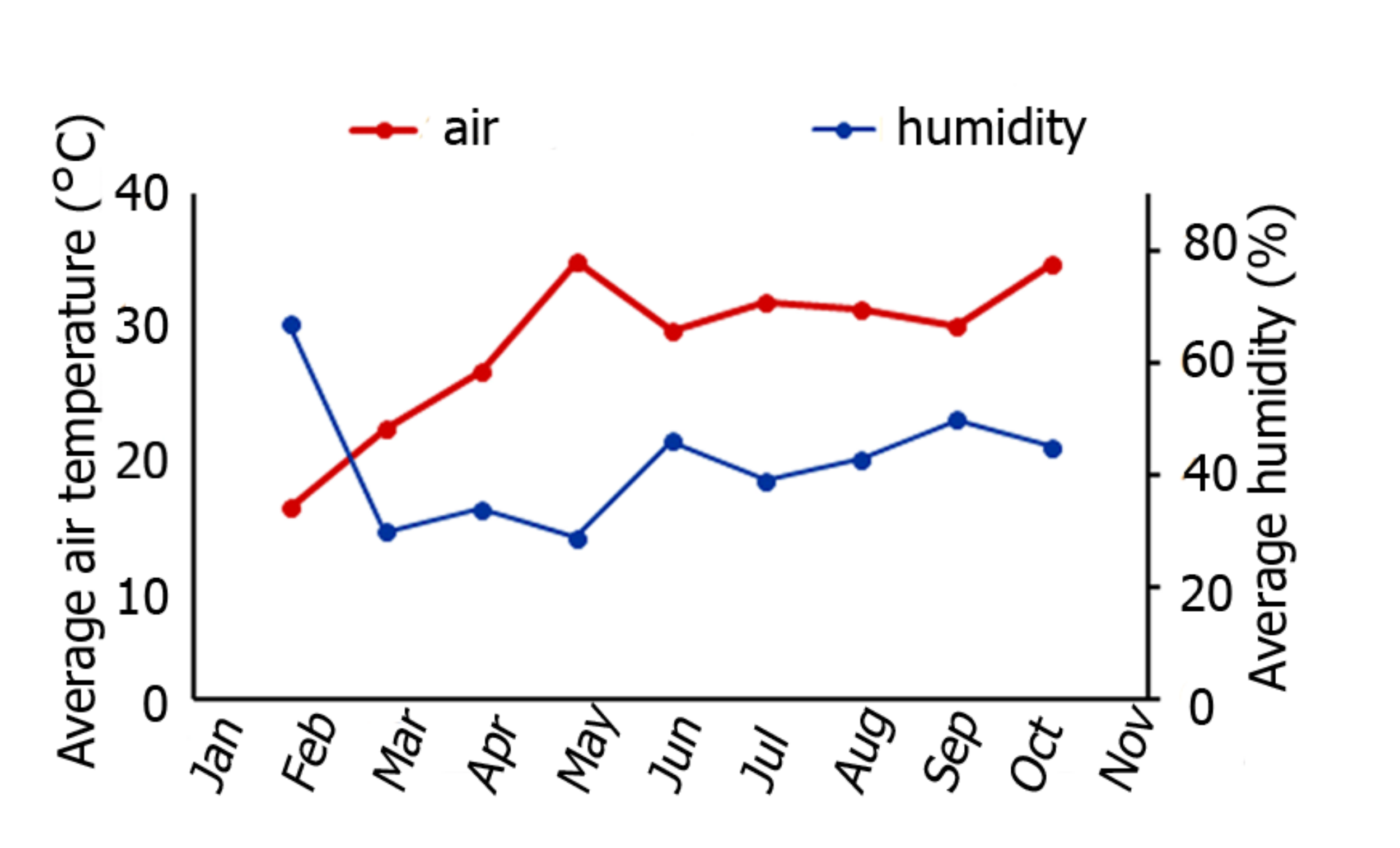


**Supplementary Figure 1.** Average air temperature and humidity measured across sampling points


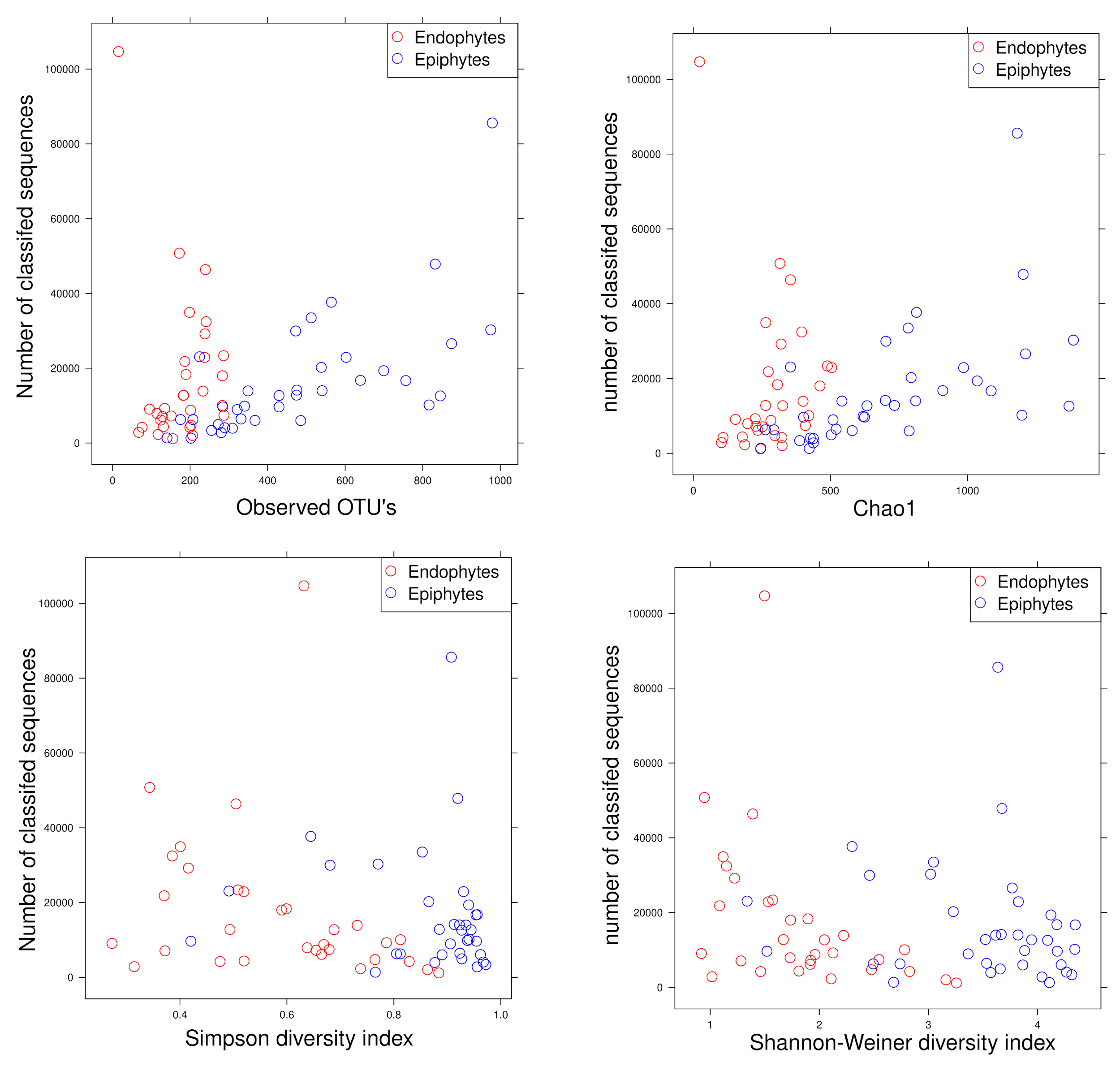


**Supplementary Figure 2.** different diversity indices in relation to the obtained number of classified sequences in each sample for both endophytes (red circles) and epiphytes (blue circles).


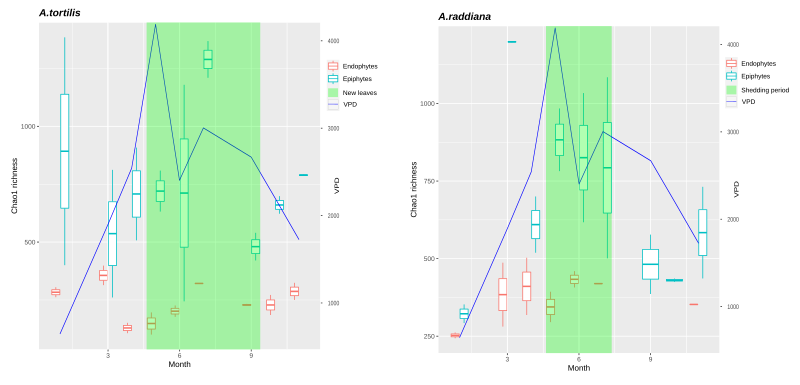
**Supplementary Figure 3.** Box plot showing Chao1 species richness for endophytic bacterial communities for A. raddiana (A) and A. tortilis, blue line showing the value of the vapour pressure deficit.


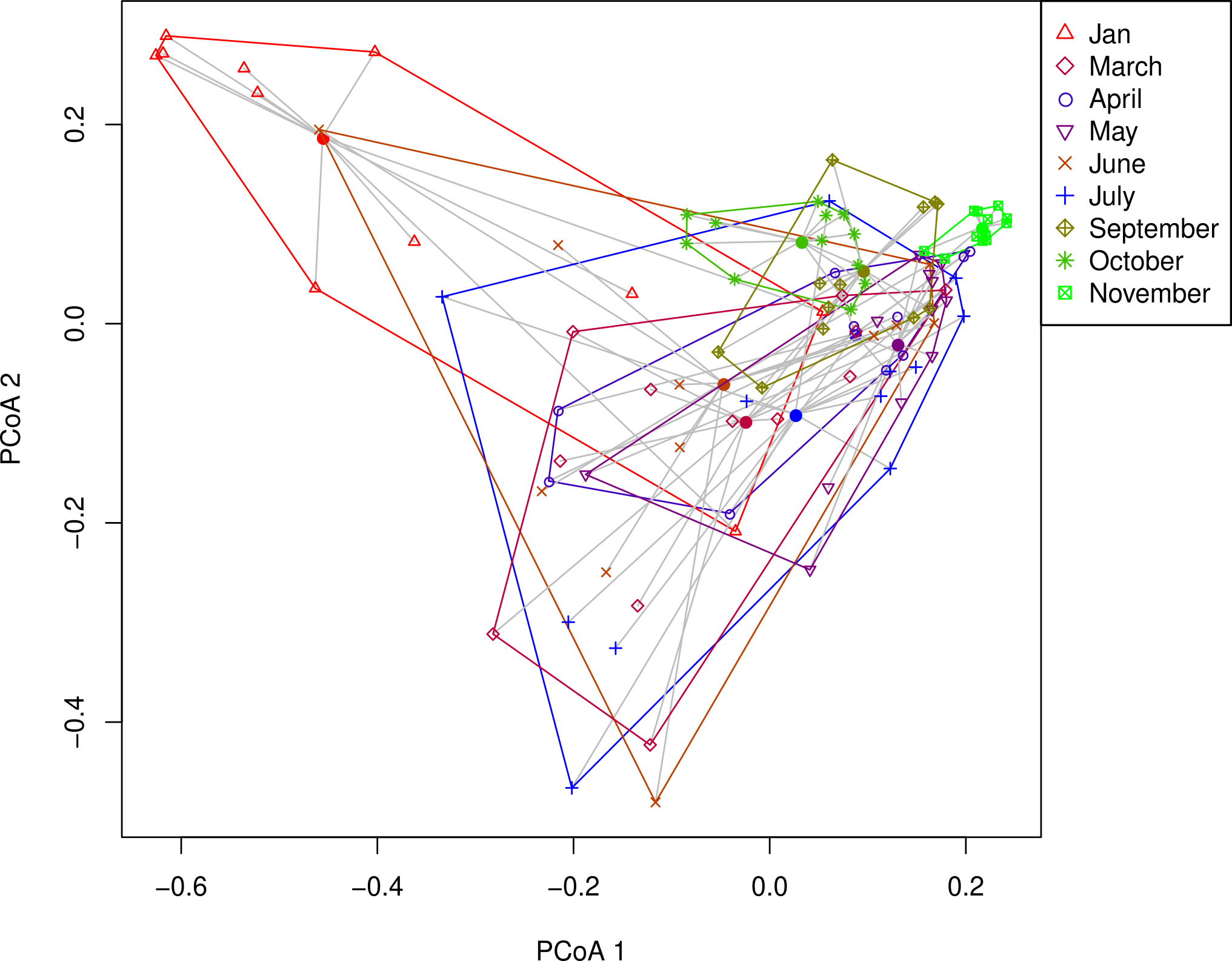

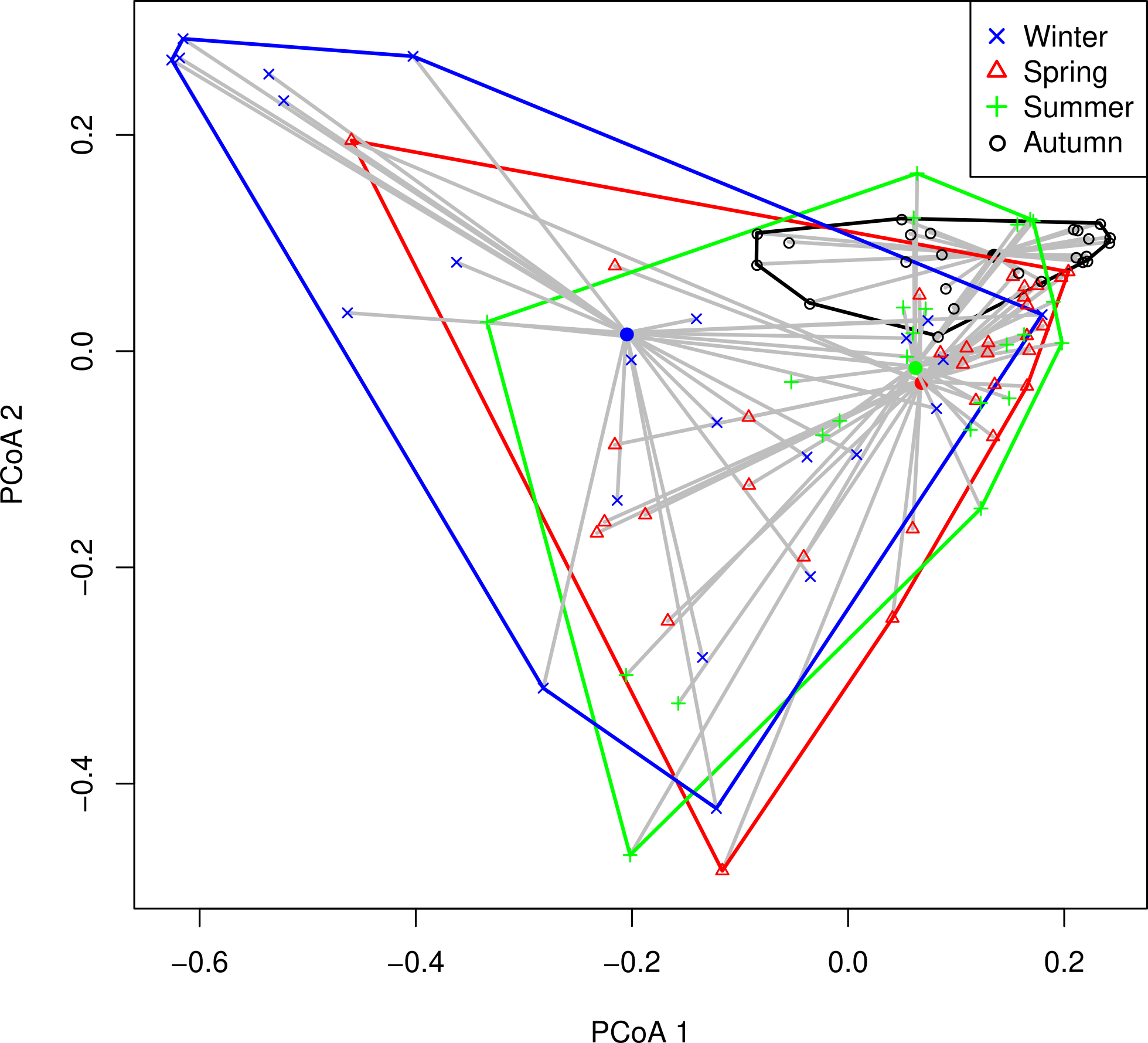


**Supplementary Figure 4.** Beta dispersion PCoA plot for epiphytic bacterial communities at different sampling month (A) and season(B).


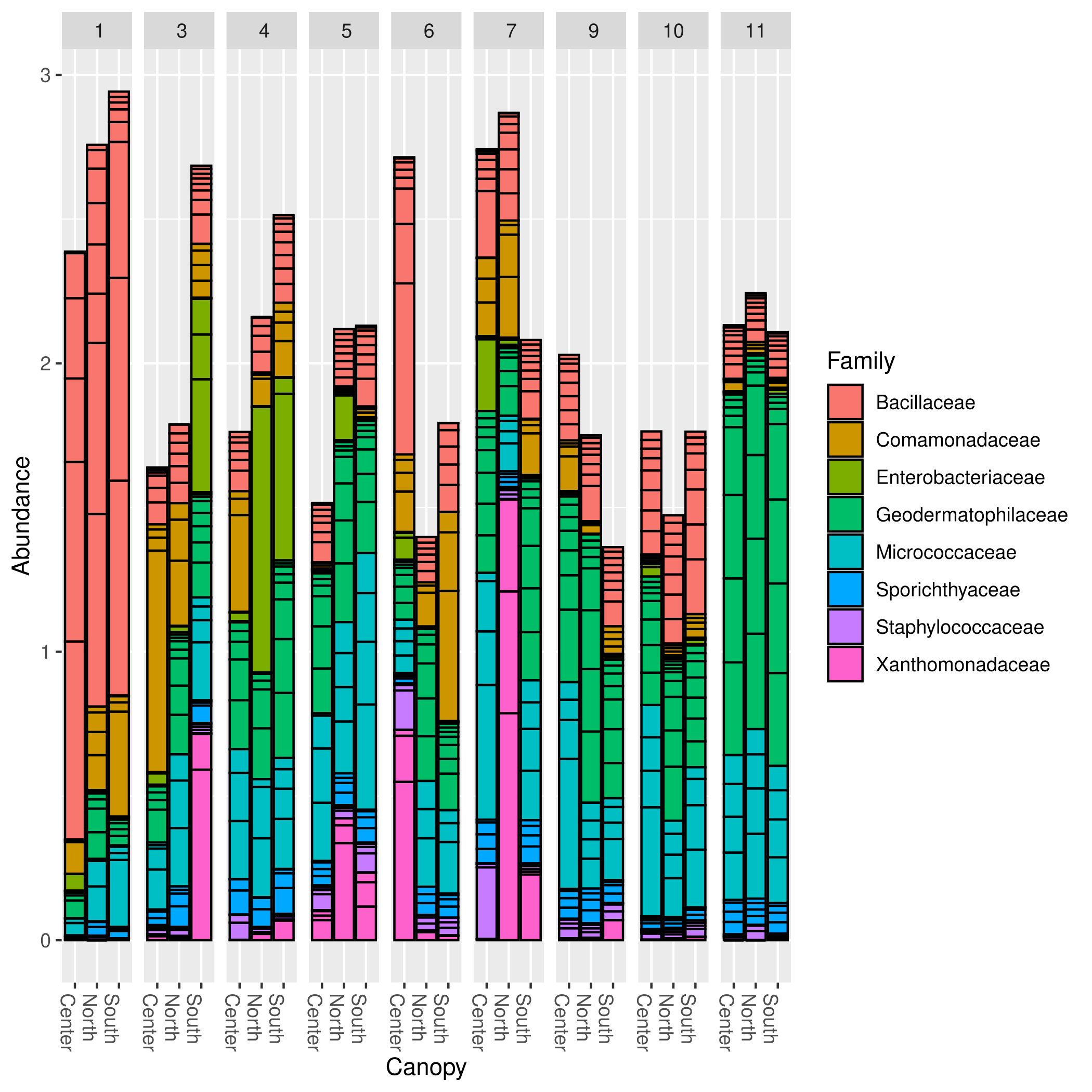


**Supplementary Figure 5.** Barplot for epiphytic bacterial families (ASV>1%) for different canopy sides at different sampling months.


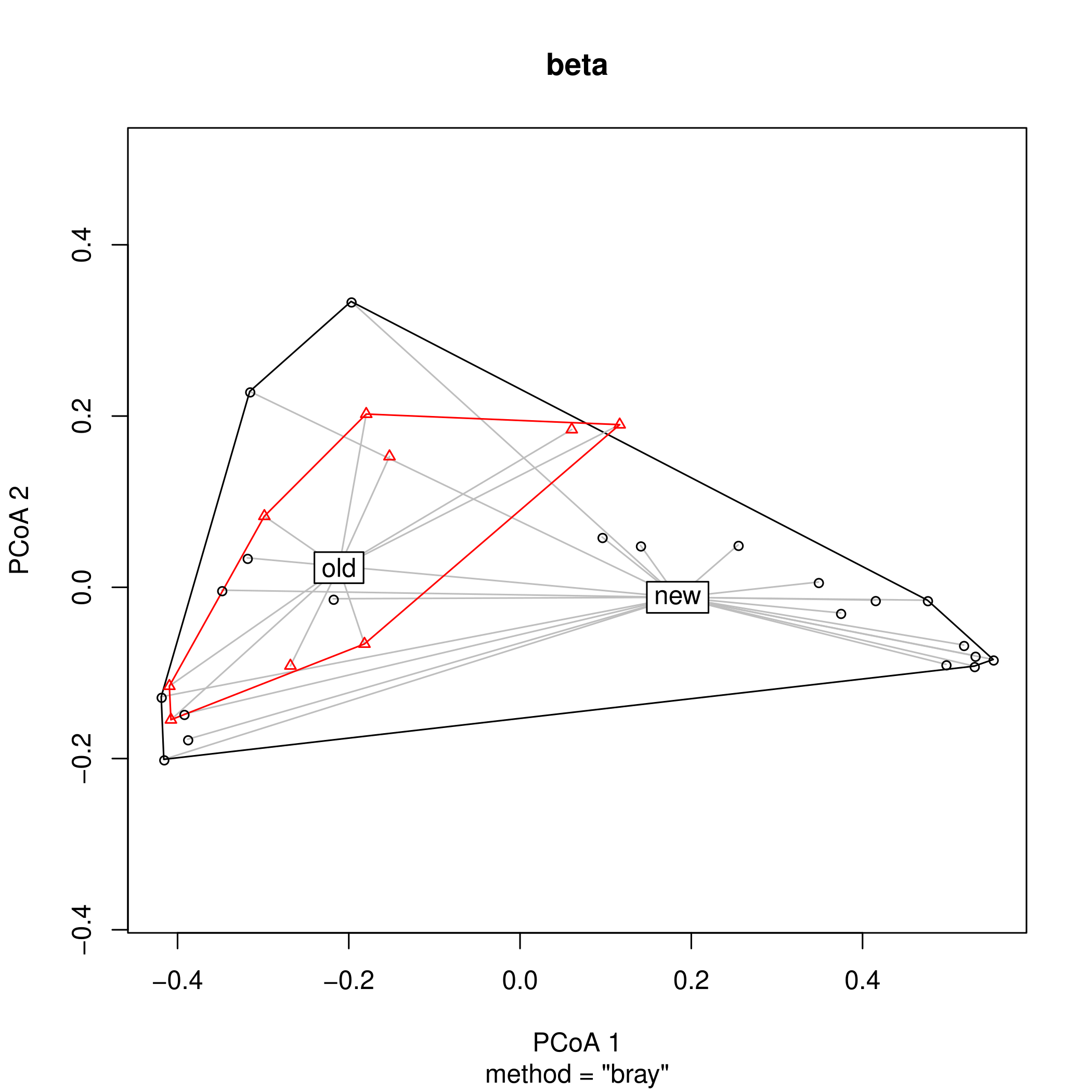

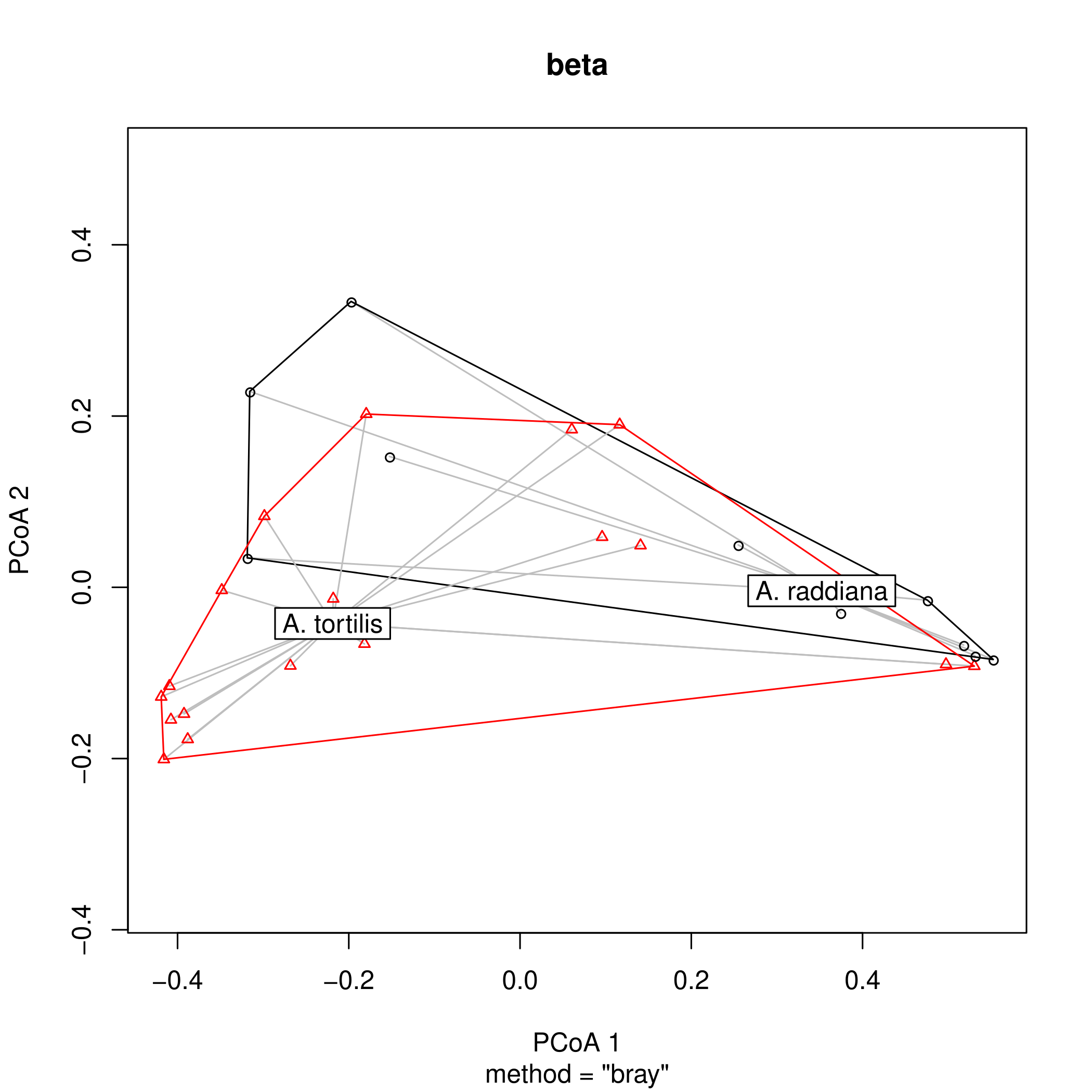


**Supplementary Figure 6.** Beta dispersion PCoA plot for endophytic bacterial communities at different tree phylogeny at shedding period “old”, non-shedding period “new” (A) and acacia species (B).


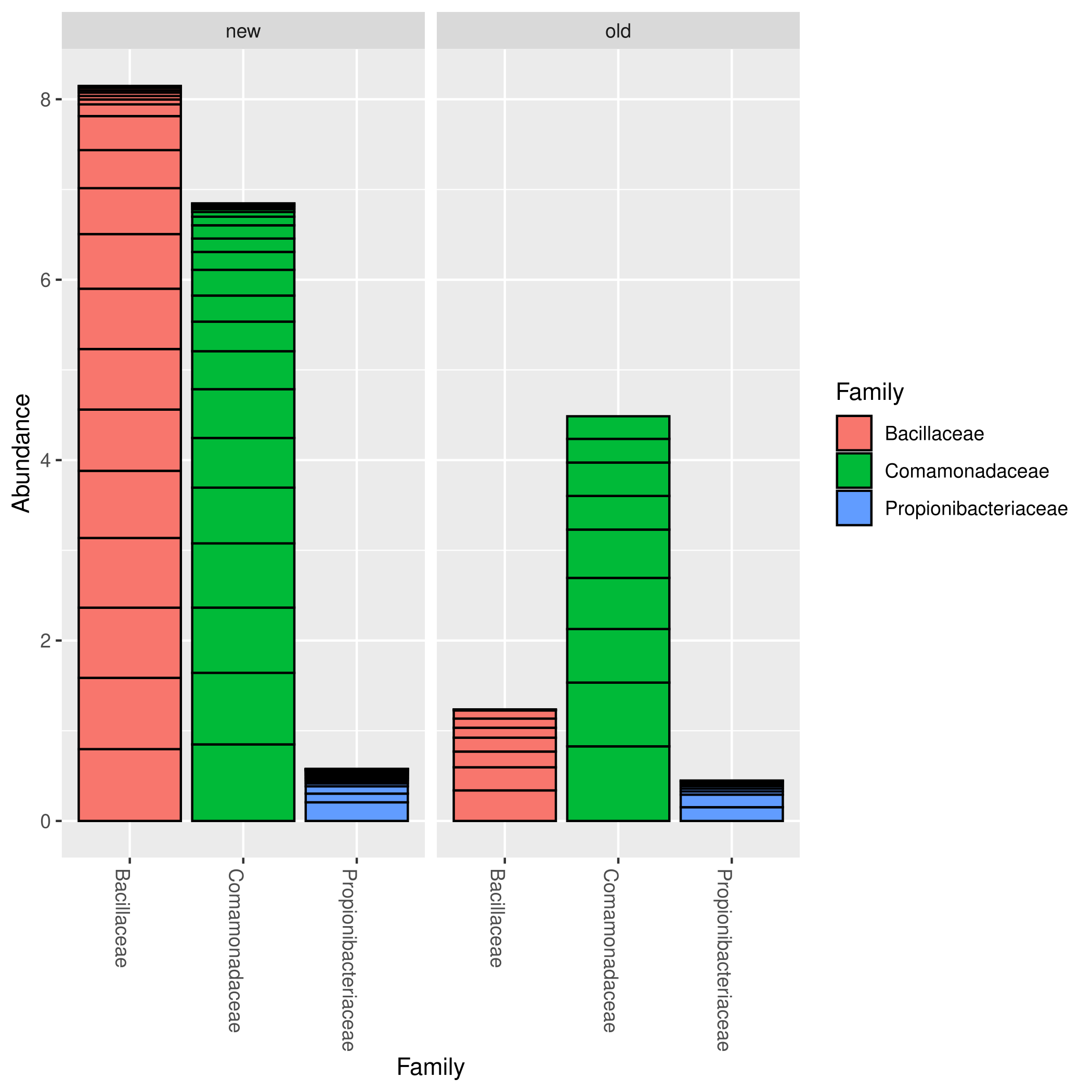

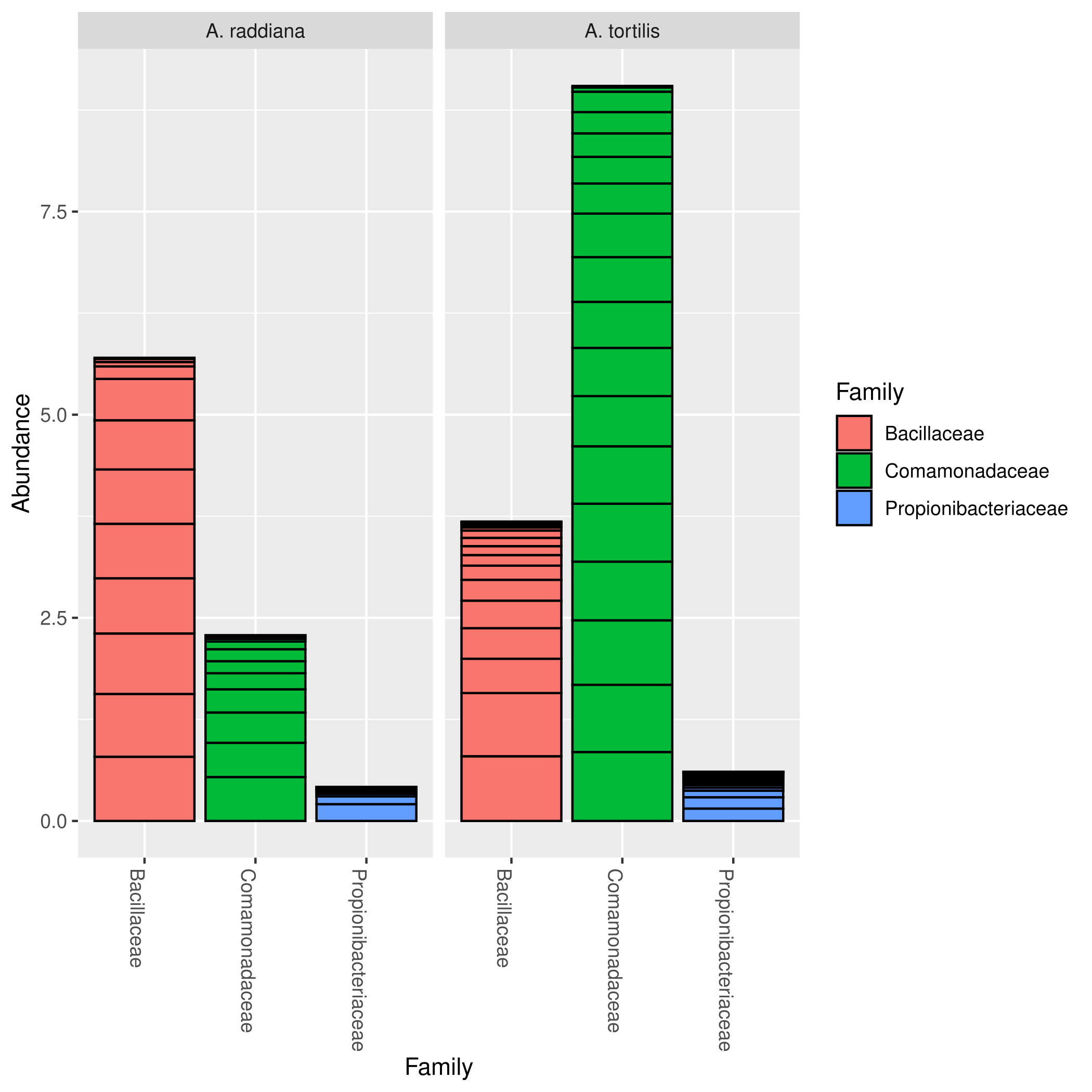
**Supplementary Figure 7.** Barplot for endophytic bacterial families (ASV>1%) for different tree phylogeny at shedding period “old”, non-shedding period “new” (A) and acacia species (B).


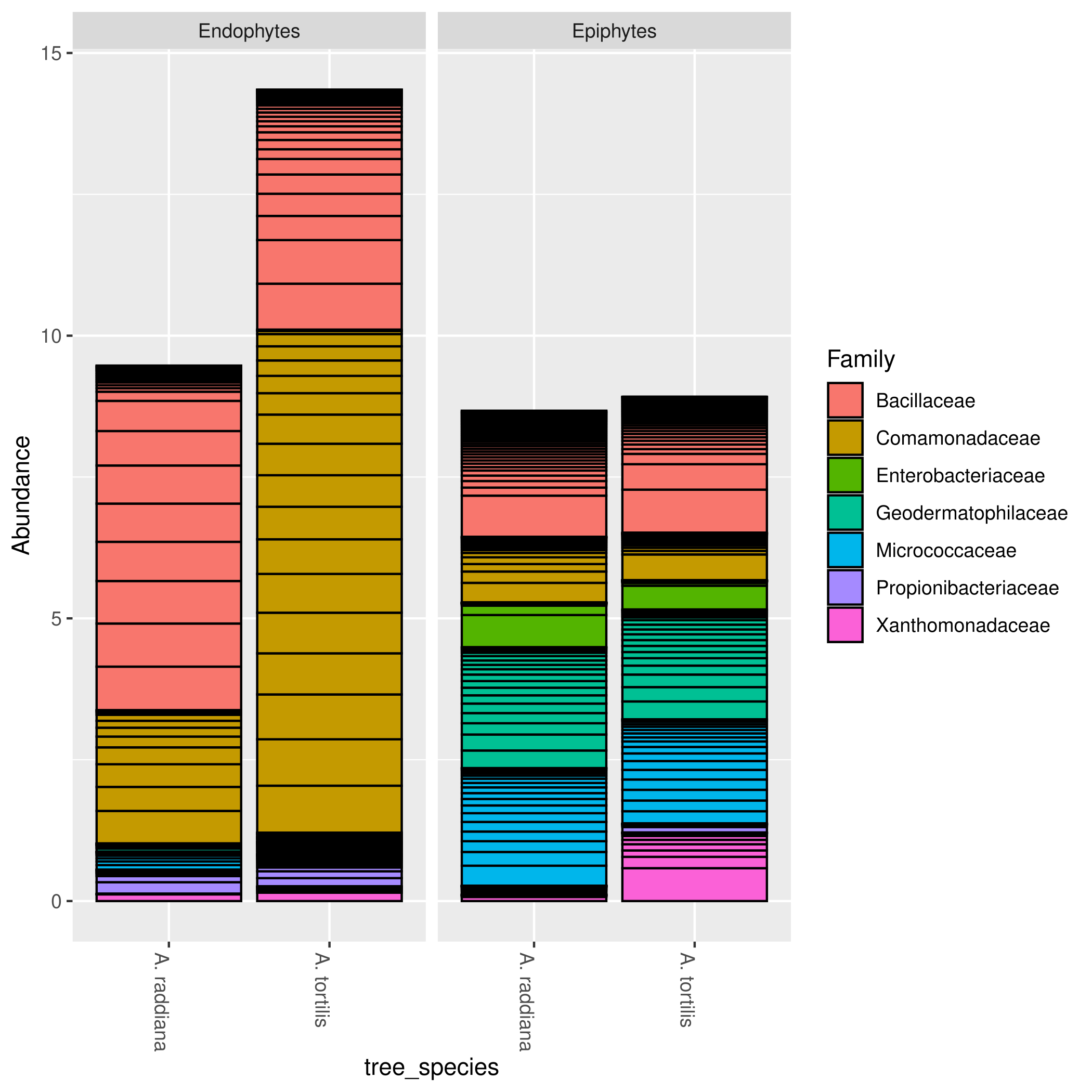


**Supplementary Figure 8.** Barplot for endophytic and epiphytic bacterial families (ASV>1%) for different acacia species.


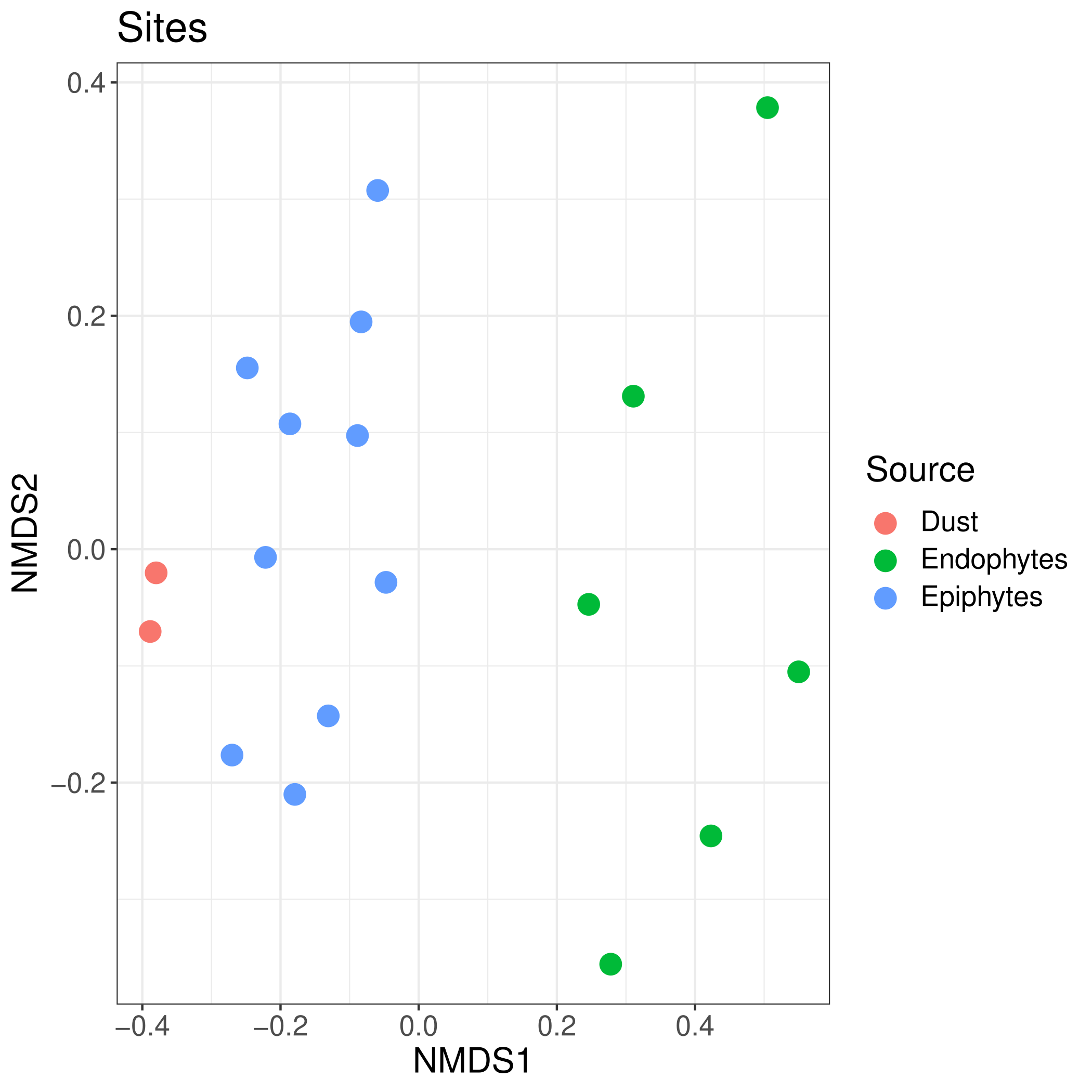

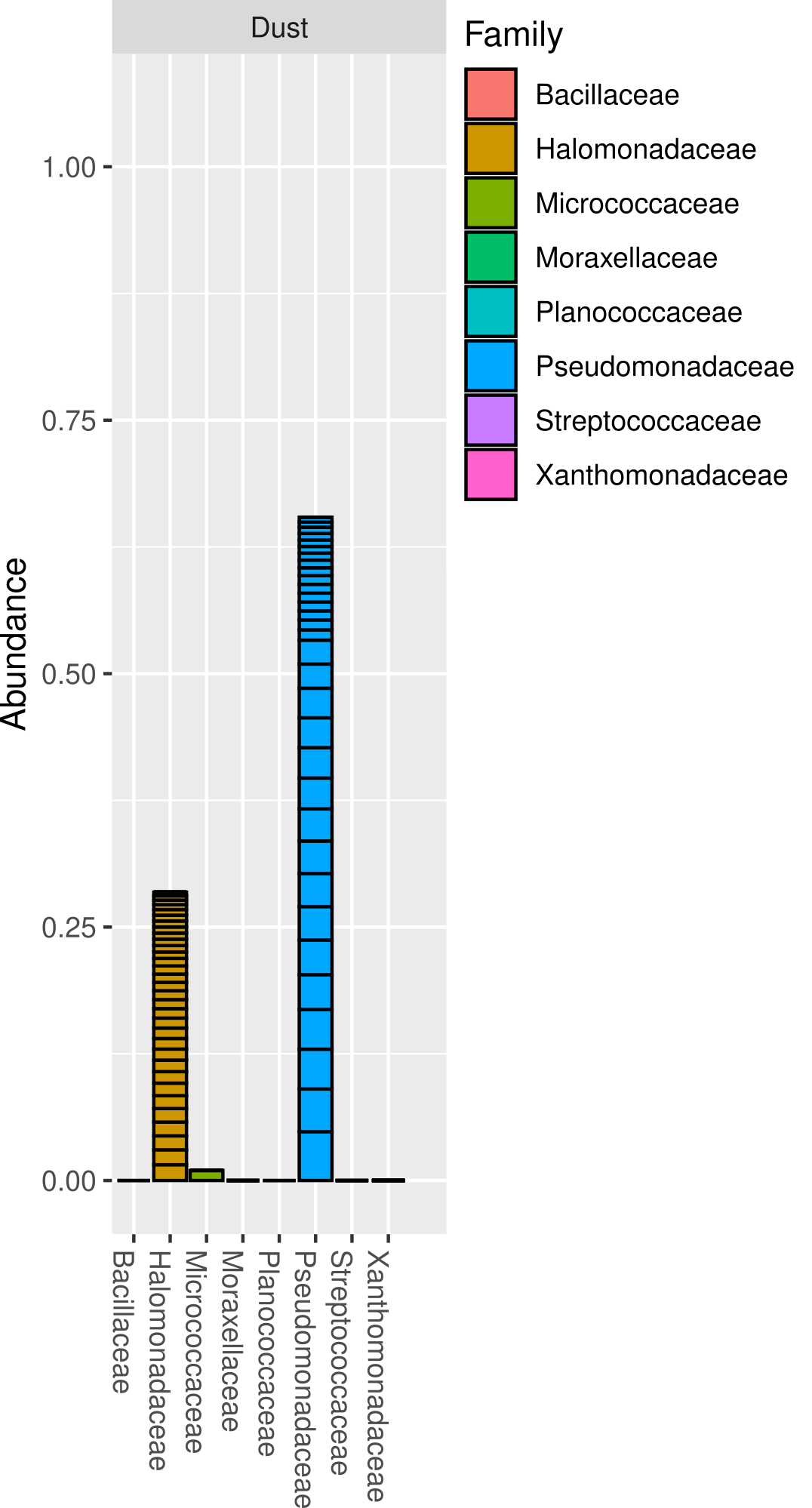

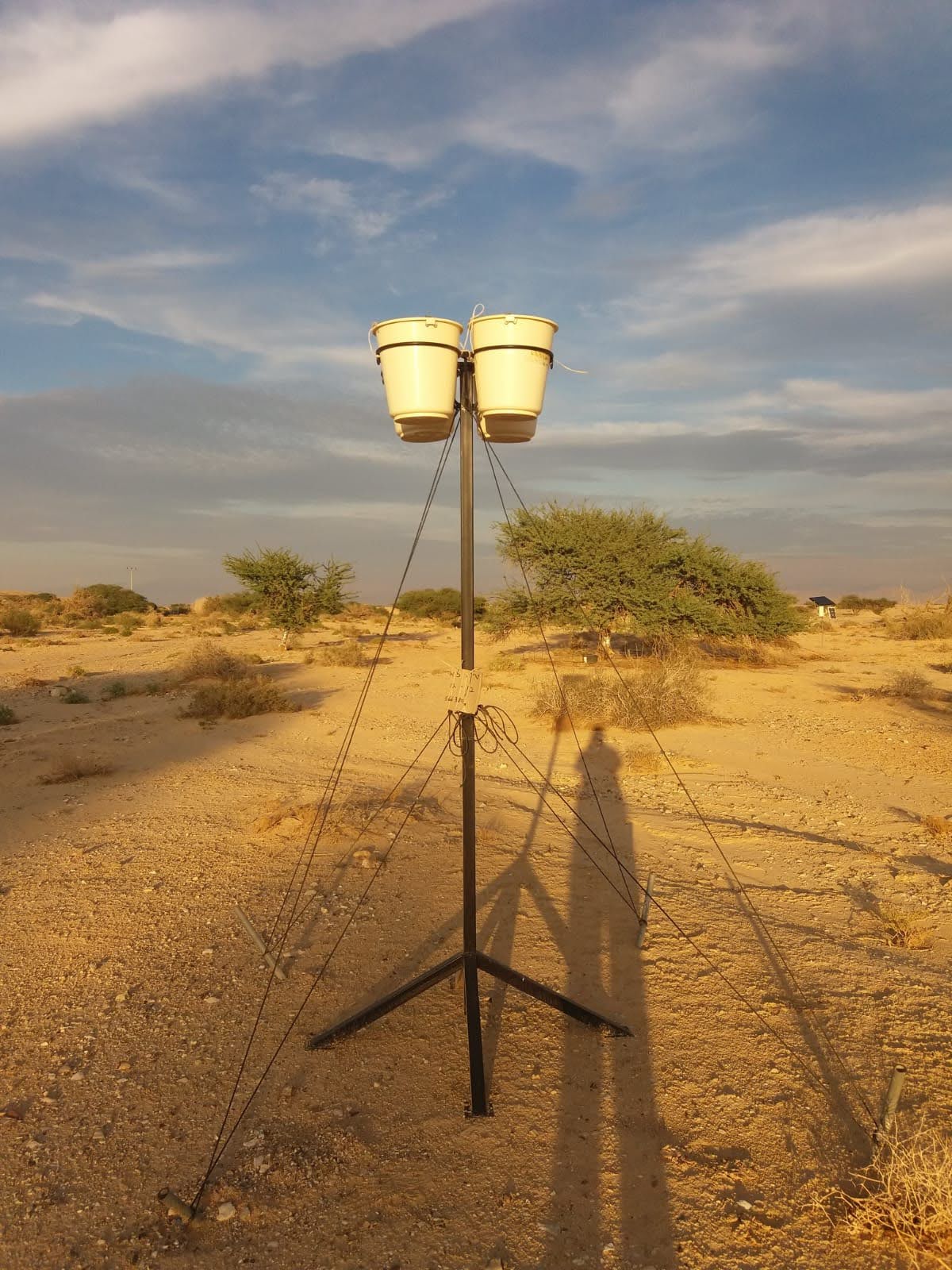


**Supplementary Figure 9:** NMDS showing epiphytes, endophytes and collected dust samples bacterial community from different experiments in 2018.

1. **Supplementary Tables**

**Supplementary Table 1.** Sampling dates and locations for the collected leaf samples at Shizaf nature reserve. Two trees of *A. raddiana* (R) and *A. tortilis* (T) were sampled for their north (N) center (C) and south (S) canopy sides as illustrated for both epi- and endophytes (only south canopy) microbial communities.

| Samples trees | Sample source | | Collection date | Samples number |
| --- | --- | --- | --- | --- |
|  | epiphytic | endophytic |  |  |
| R284, R286, T23, T300 | N, C and S | S | 24/01/2015 | 16 |
| R284, R286, T23, T300 | N, C and S | S | 24/03/2015 | 16 |
| R284, R286, T23, T300 | N, C and S | S | 20/04/2015 | 15 (sample R286_epi_N) was lost in processing |
| R284, R286, T23, T300 | N, C and S | S | 28/05/2015 | 16 |
| R284, R286, T23, T300 | N, C and S | S | 10/06/2015 | 16 |
| R284, R286, T23, T300 | N, C and S | S | 11/07/2015 | 15 (sample R286_endo_S) was lost in processing |
| R284, R286, T23, T300 | N, C and S | S | 30/09/2015 | 14 (R284_ and R286_endo_S) was lost in processing |
| R284, R286, T23, T300 | N, C and S | S | 18/10/2015 | 14 (R284_ and R286_endo_S) was lost in processing |
| R284, R286, T23, T300 | N, C and S | S | 16/11/2015 | 15 (R284_endo_S) was lost in processing |

**Supplementary Table 2.** The 16S rDNA primer sets used in this study in PCR I. Shown are the sequences of each primer pair (with their working concentrations), product length in base pairs (bp), the average (±SD) numbers of raw sequences obtained after sequencing and after classification, alongside the number of final samples after sequence classification and curation (n). The source of the primers is also listed.

| Primer  pair # | Primer sequences (3`-5`) [Concentration] | Product length (bp) | Raw sequences | Classified sequences | Reference |
| --- | --- | --- | --- | --- | --- |
| 1 | Forward_primer (F70): TGGCGAACGGGTGAGTAA [10 µM]  Reverse_primer (R307): GACGTGTGCTCTTCCGATCTCCGTGTCTCAGTCCCARTG [20 µM] | 237 | 16,286±13,913 (n=134) | 6,067±7,562 (n=111) | Fuks et al., 2018 |
| 2 | Forward_primer (F310): CTCCTACGGGAGGCAGC [10 µM]  Reverse_primer (R499): AGACGTGTGCTCTTCCGATCTGTATTACCGCGGCTGCTG [10 µM] | 189 | 2,442±3,361 (n=49) | 1,847±1,380 (n=10) | Fuks et al., 2018 |
| 3 | Forward_primer (F649): GTGTAGCGGTGRAATGCG [20 µM]  Reverse_primer (R889): AGACGTGTGCTCTTCCGATCTCCCGTCAATTCMTTTGAGTT [20 µM] | 240 | 38,683±18,723 (n=137) | 15,424±13,784 (n=137) | Fuks et al., 2018 |
| 4 | Forward_primer (F908): GGAGCATGTGGWTTAATTCGA [20 µM]  Reverse_primer (R1067): AGACGTGTGCTCTTCCGATCTCGTTGCGGGACTTAACCC [10 µM] | 159 | 8,128±8,493 (n=127) | 2,402±4,563 (n=74) | Fuks et al., 2018 |
| 5 | Forward_primer (F1139): GGAGGAAGGTGGGGATGAC [10 µM]  Reverse_primer (R1364): AGACGTGTGCTCTTCCGATCTAAGGCCCGGGAACGTATT [10 µM] | 225 | 2,864±3,833 (n=36) | 3,085±3,547 (n=11) | Fuks et al., 2018 |

**Supplementary Table 3.** The position of each primer, sequence and working concentration of each 16S rDNA amplicon used in PCR-II.

| Pair # | Sequence (3`-5`) [Concentration] |
| --- | --- |
| 1^st^ Forward primer | AATGATACGGCGACCACCGAGATCTACACTCTTTCCCTACACGACGCTCTTCCGATCTTGGCGAACGGGTGAGTAA [4 µM] |
| 2^nd^ Forward primer | AATGATACGGCGACCACCGAGATCTACACTCTTTCCCTACACGACGCTCTTCCGATCTACTCCTACGGGAGGCAGC [4 µM] |
| 3^rd^ Forward primer | AATGATACGGCGACCACCGAGATCTACACTCTTTCCCTACACGACGCTCTTCCGATCTGTGTAGCGGTGRAATGCG [8 µM] |
| 4^th^ Forward primer | AATGATACGGCGACCACCGAGATCTACACTCTTTCCCTACACGACGCTCTTCCGATCTGGAGCATGTGGWTTAATTCGA [8 µM] |
| 5^th^ Forward primer | AATGATACGGCGACCACCGAGATCTACACTCTTTCCCTACACGACGCTCTTCCGATCTGGAGGAAGGTGGGGATGAC [4 µM] |
| Reverse primer | CAAGCAGAAGACGGCATACGAGAT*NNNNNNNN*GTGACTGGAGTTCAGACGTGTGCTCTTCCGATCT [8 µM] |

**Supplementary Table 4.** Different diversity estimates (A-D) during different sampling months for A. raddiana and A. tortilis for both endo end epiphytes

**(A)** Chao1 index

| Month | A. Raddiana | | A. tortilis | |
| --- | --- | --- | --- | --- |
|  | Endophytes | Epiphytes | Endophytes | Epiphytes |
| January | 252.8 | 322.4 | 283.5 | 892.4 |
| March | 384.0.9 | 11.8 | 356.1 | 536.4 |
| April | 410.6 | 609.7 | 84.5 | 707.8 |
| May | 344.3 | 882.8 | 148.3 | 720.2 |
| June | 433.4 | 825.4 | 201.5 | 711.7 |
| July | 419.5 | 792.7 | 321.5 | 1289.0 |
| September | - | 481.8 | 228.6 | 480.3 |
| October | - | 430.4 | 228.4 | 660.4 |
| November | 352.4 | 583.8 | 387.0 | 789.1 |

**(B)** Number of observed OTU’s

| Month | A. Raddiana | | A. tortilis | |
| --- | --- | --- | --- | --- |
|  | Endophytes | Epiphytes | Endophytes | Epiphytes |
| January | 175.5 | 114.5 | 185 | 628.5 |
| March | 242.5 | 823 | 201.5 | 368.5 |
| April | 236.5 | 400.5 | 84.5 | 479 |
| May | 220.5 | 556 | 89.5 | 483.5 |
| June | 284 | 518.5 | 140 | 558.5 |
| July | 282 | 513 | 202 | 858.5 |
| September | - | 310 | 128 | 274.5 |
| October | - | 283.5 | 150.5 | 451 |
| November | 238 | 390.5 | 154.5 | 510.5 |

**(C)** Phylogenetic diversity

| Month | *A. Raddiana* | | *A. tortilis* | |
| --- | --- | --- | --- | --- |
|  | Endophytes | Epiphytes | Endophytes | Epiphytes |
| January | 9.4 | 11.9 | 10.1 | 23.9 |
| March | 11.9 | 28.9 | 9.3 | 15.0 |
| April | 10.5 | 18.1 | 6.0 | 20.9 |
| May | 10.7 | 17.4 | 5.5 | 20.1 |
| June | 13.8 | 21.5 | 8.4 | 16.6 |
| July | 14.8 | 20.2 | 12.3 | 31.2 |
| September | - | 15.0 | 7.9 | 14.2 |
| October | - | 14.5 | 9.2 | 19.0 |
| November | 7.7 | 18.7 | 9.9 | 22.5 |

(D) Shannon-Wiener diversity index

| Month | A. Raddiana | | A. tortilis | |
| --- | --- | --- | --- | --- |
|  | Endophytes | Epiphytes | Endophytes | Epiphytes |
| January | 2.18 | 2.03 | 1.78 | 2.27 |
| March | 1.76 | 4.00 | 1.58 | 2.39 |
| April | 1.37 | 2.99 | 1.19 | 3.77 |
| May | 1.81 | 3.43 | 1.37 | 3.88 |
| June | 2.14 | 4.00 | 1.86 | 3.15 |
| July | 2.78 | 4.00 | 2.99 | 3.92 |
| September | - | 4.26 | 2.02 | 3.86 |
| October | - | 4.15 | 1.59 | 3.92 |
| November | 1.38 | 3.54 | 1.66 | 3.54 |
